# DNA Repair Deficiency Regulates Immunity Response in Cancers: Molecular Mechanism and Approaches for Combining Immunotherapy

**DOI:** 10.3390/cancers15051619

**Published:** 2023-03-06

**Authors:** Yi Xu, Somaira Nowsheen, Min Deng

**Affiliations:** 1 State Key Laboratory of Molecular Oncology and Department of Radiation Oncology, National Cancer Center/National Clinical Research Center for Cancer/Cancer Hospital, Chinese Academy of Medical Sciences and Peking Union Medical College, Beijing 100021, China; 2 Department of Dermatology, University of California San Diego, San Diego, CA 92122, USA

**Keywords:** DNA damage response, cancer therapy, immunotherapy, cell death, biomarker, tumor microenvironment, DNA repair, cell death

## Abstract

**Simple Summary:**

DNA repair pathways play a crucial role in maintaining the stability of a cell’s genetic material. When these pathways are defective, it can lead to genomic instability in cancer cells, which can increase their ability to stimulate an immune response. Inhibiting DNA damage response, the process that helps repair DNA damage, has been shown to increase the effectiveness of anticancer immunotherapies. In this review, we will explore how deficits in the DNA repair pathway can affect the immune system’s ability to fight cancer. We will also examine clinical trials that have combined inhibition of DNA damage response with immune-oncology treatments. A better understanding of these pathways could help improve the effectiveness of cancer immunotherapies and other treatments for various types of cancer.

**Abstract:**

Defects in DNA repair pathways can lead to genomic instability in multiple tumor types, which contributes to tumor immunogenicity. Inhibition of DNA damage response (DDR) has been reported to increase tumor susceptibility to anticancer immunotherapy. However, the interplay between DDR and the immune signaling pathways remains unclear. In this review, we will discuss how a deficiency in DDR affects anti-tumor immunity, highlighting the cGAS-STING axis as an important link. We will also review the clinical trials that combine DDR inhibition and immune-oncology treatments. A better understanding of these pathways will help exploit cancer immunotherapy and DDR pathways to improve treatment outcomes for various cancers.

## 1. Introduction

Cancer has become a leading cause of death in many countries and is still a major public health problem worldwide [1]. The classical and primary therapies are surgery, radiotherapy, and chemotherapy. Along with a better understanding of the molecular biology of the tumor cells, molecularly targeted therapies are designed to inhibit a target that is abnormal in malignant tissues when compared with normal tissues [2,3]. In comparison, most target drugs have shown limited efficacy against solid tumors, largely due to the fact that tumors frequently develop resistance to these therapies [4]. In recent years, immunotherapy has had remarkable clinical success, including immune checkpoint blockade (ICB) and adoptive cell therapy. The antibodies targeting programmed cell death 1 (PD1), PD1 ligand 1 (PDL1), and cytotoxic T-lymphocyte-associated protein 4 (CTLA4) as ICBs have been approved for broad application to treat solid tumors [5]. Anti-PD therapy dominates ICB therapies and has been shown to be superior to anti-CTLA4 therapy in a wide variety of tumors [6,7]. However, the response rate of anti-PD therapy alone is usually only 20% in advanced-stage cancers, and adaptive immune resistance mechanisms also help cancer cells to escape attacks by the immune system. Thus, combining immunotherapy with other approaches to improve the anti-tumor effect is reasonable. Researchers have proposed the promising approach of utilizing DNA repair deficiency to enhance anti-tumor immunity [8].

The DNA damage response (DDR) is essential for maintaining genomic stability by repairing different types of DNA damage [9]. Cancer cells with high underlying levels of DNA damage are more dependent on DDR for survival when compared to normal cells [10]. Deficiencies in DDR result in the accumulation of DNA damage and enhance immunogenicity in tumors. Numerous studies have identified that DNA damage agents modify systemic immune functions [11,12,13]. In addition, clinical data show that a loss of mismatch repair could be a predictive biomarker for ICB response [14]. Thus, combining DDR network inhibitors with immunotherapy attracts more attention to clinical testing.

Here, we review the mechanism of DDR and discuss its interactions with anti-tumor immunotherapy. We also present the clinical implications of DDR, including combination with immune-oncology treatment in clinical trials and immune response prediction as a biomarker. Finally, we evaluate the opportunities and development of DDR-immunotherapy combinations in anticancer therapies.

## 2. DNA Damage and Repair Pathway

DNA injuries occur as a result of intrinsic or extrinsic agents and can include modifications to bases and sugars, single- and double-strand breaks (SSBs, DSBs), DNA-protein crosslinks, and base-free sites [15]. While some specific DNA lesions can lead to mutations that cause cancer, the main consequence of DNA injuries is the threat they pose to DNA integrity and stability [16]. To prevent accumulated DNA lesions from causing irreversible harm, cells initiate DDR, which senses the DNA damage, signals its presence, and mediates its repair. DDR kinases, including DNA-dependent protein kinase (DNA-PK), ataxia telangiectasia mutated (ATM), and ataxia telangiectasia and Rad3-related (ATR), are activated at DNA lesions, which then mediate cell cycle arrest and DNA repair [17].

**Figure 1:**
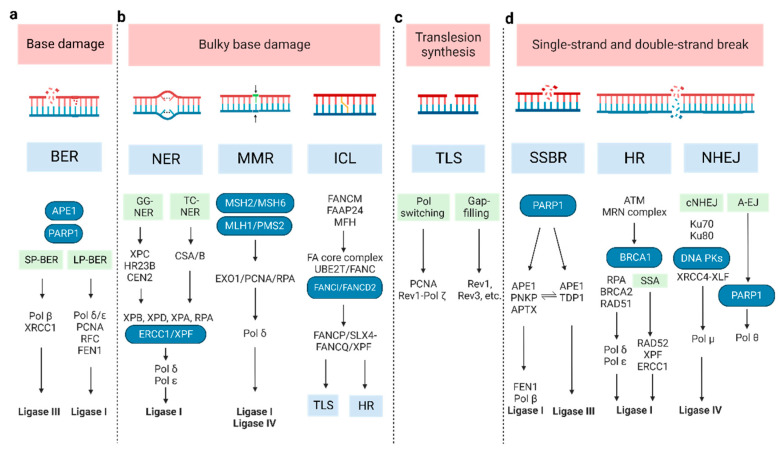
The molecular pathways of DNA damage repair. (**a**) BER can remove a single damaged base and is divided into two sub-pathways: short-patch (SP-BER) and long-patch (LP-BER). (**b**) For bulky base damage, NER removes the damaged base and several adjacent nucleotides, through global genome NER (GG-NER) and transcription-coupled NER (TC-NER); MMR corrects mis-incorporated bases and strand crosslinks; ICL repair, also known as FA pathway, resolves the covalently linked DNA strands. (**c**) TLS repair uses specialized DNA Pols to bypass DNA damage or fill single-strand DNA gaps by inserting and/or extending nucleotides, via Pol switching model and the gap-filling model. (**d**) SSBR repair shares most enzymatic steps with BER pathway; the main repair processes for DSB are HR, SSA, cNHEJ, and A-EJ. (A-EJ: alternative end joining; BER: base excision repair; cNHEJ: classic non-homologous end joining; DSB: double-strand break; HR: homologous recombination; ICL: inter-strand crosslink; MMR: the Mismatch Repair; NER: nucleotide excision repair; NHEJ: non-homologous end joining; SSBR: single-strand break repair; TLS: Trans lesion synthesis).

In the cell cycle arrest pathway, ATM and DNA-PK are mainly activated by DSBs, while ATR is activated by SSBs. These kinases phosphorylate downstream cell cycle checkpoint kinases. The active CHK1 and CHK2 then phosphorylate p53, CDC25, and WEE1, which increases the expression of p21 (p53), inhibits CDK activity and leads to cell cycle arrest at G1/S and G2/M transition (CDC25 and WEE1) [9,18]. In addition, the molecular pathways of primary DNA repair mechanisms that function in common types of DNA damage are introduced below (Figure 1) [19,20,21].

### 2.1. Base Damage Repair

Base excision repair (BER): Base damage occurs when chemical bonds within the DNA molecule are formed abnormally. BER can remove a single damaged base. At the beginning of BER, a series of lesion-specific DNA glycosylases remove the damaged base by cleaving the N-glycosidic bond linking the base to its corresponding deoxyribose [22]. Apurinic/apyrimidinic endonuclease 1 (APE1) and poly ADP-ribose polymerase 1 (PARP1) can sense and bind to the damage site. This catalyzes poly ADP-ribosylation (PAR) and some other protein substrates, which allows for the recruitment of repair proteins. The next synthesis/ligation step of BER is divided into two sub-pathways—short-patch and long-patch [23]. In short-patch BER, the polymerase beta (Pol β) fills the generated gap with the correct nucleotide [24]. The successive ligation of the DNA ends demands either DNA ligase I (LIG1) or the complex of DNA ligase III (LIG3) and X-ray repair cross-complementing protein 1 (XRCC1). In long-patch BER, proliferating cell nuclear antigen (PCNA), replication factor-C (RFC), flap endonuclease-1 (FEN1), Pol δ/ε, and LIG1 are included [24].

### 2.2. Bulky Base Damage Repair

Nucleotide excision repair (NER): This pathway removes bulky lesions, which involves removing the damaged base and several adjacent nucleotides [25]. The significant lesions initiating NER are pyrimidine dimers, such as cyclobutene pyrimidine dimers (CPD), and 6–4 photo-products induced by ultraviolet light- cisplatin-DNA intra-strand crosslinks [26,27]. In the recognition step, there are two different pathways, termed global genome NER (GG-NER) and transcription-coupled NER (TC-NER), whose recognition factor is the XPC/HR23B/CEN2 (XP complementation group C/Rad23 homolog B/Centrin-2) protein complex and CSA/B (Cockayne syndrome A and B, displacing the stalled RNA polymerase II), respectively [28,29]. The following excision and polymerization steps are all the same. XPB and XPD orchestrate the asymmetric unwinding of the DNA helix, accompanied by XPA and RPA binding to the damaged region. Then, the structure-specific endonucleases XPG and XPF/ERCC1 lead to nucleotide excision. Lastly, the resulting gap is resynthesized by Pol δ/ε and sealed by LIG1 [30].

Mismatch repair (MMR): This corrects mis-incorporated bases and strand crosslinks that occur during DNA replication. Defective MMR (dMMR) causes microsatellite instability (MSI) and an increased mutation frequency, which increases the risk of certain cancers such as Lynch syndrome and colon cancer. The MLH/MSH/PMS gene family plays a critical role in MMR [31,32]. The MSH2-MSH6 heterodimer preferentially recognizes base-base mismatches and small insertion/deletion loops (IDLs), while the MSH2-MSH3 heterodimer recognizes larger IDLs. MLH1 and PMS2, which contain the primary endonuclease activity (~90%), facilitate downstream events. The degradation of the error-containing strand is performed by Exo1 [32,33]. Then, polymerized DNA (synthesized by Pol δ), accompanied by PCNA and RPA, resynthesizes a vast gap, and LIG1 or LIG4 seals the remaining nick [34].

Inter-strand crosslink (ICL) repair: ICLs are a form of DNA damage in which two complementary DNA strands are covalently linked. To resolve ICLs, Fanconi Anemia (FA) proteins are primarily involved during the S phase of the cell cycle [35,36]. FANCM and its interacting partners (FAAP24 and MFH) recognize the lesions and recruit the FA core complex and UBE2T/FANC, to monoubiquitinate the ID2 complex (FANCI and FANCD2 heterodimer) [37,38,39]. Then, the monoubiquitinated central complex activates FANCP/SLX4-FANCQ/XPF to unhook ICLs, generating different types of lesions. These ICL-associated lesions are repaired by other DNA repair pathways, including translesion synthesis (TLS) and homologous recombination (HR) [35,40,41].

### 2.3. Translesion Synthesis

TLS repair: TLS, an DNA damage tolerance mechanism, uses specialized DNA Pols to bypass DNA damage or fill single-strand DNA (ssDNA) gaps by inserting and/or extending nucleotides [42]. It can be error-prone or error-free. Two models have been proposed to explain TLS: the Pol switching model and the gap-filling model [21,43]. In the former, the inserter TLS enzyme (usually a Pol h, Pol i, or Pol j), which incorporates a nucleotide opposite the DNA lesion, is replaced by extender TLS enzyme (usually Pol ζ (REV3 and REV7), in some cases by Pol j) [44,45]. The Rev1-Pol ζ complex is the most efficient among TLS Pols [44,45,46,47], initiated by monoubiquitinated PCNA [46,48]. In the latter, TLS polymerases (Rev1, Rev3, etc.) repair ssDNA to protect cells from replication stress, though the exact order of events is still unknown [49,50]. The TLS pathway has also been implicated in other DDRs, including HR, NER, and non-homologous end joining (NHEJ) [51,52,53,54].

### 2.4. SSB and DSB Break Repair

SSB repair (SSBR): SSBs arise either directly or indirectly (e.g., during BER of base damage) [55]. Therefore, SSBR shares several enzymatic steps with the BER pathway. In the long-patch SSBR pathway, SSBs are detected by PARP1, following end processing by APE1/PNKP (poly-nucleotide kinase 30-phosphate)/APTX (aprataxin). Next, FEN1 removes the damaged termini, following which Pol β and LIG1 repair the gap [56,57,58,59]. Different from this, APE1 recognizes the lesion and LIG3 catalyzes ligation in the short-patch SSBR pathway, while TDP1 (tyrosyl-DNA phosphodiesterase 1) executes the end-processing function in the TOP1-SSB pathway [60,61].

DSBs repair: The main processes are HR, single-strand annealing (SSA), classical NHEJ (cNHEJ), and alternative end joining (A-EJ) [62,63,64,65]. HR repair is mostly error-free [66] and only happens during the S phase and subsequent G2/M phases [67]. Firstly, the Mre11-Rad50-Nbs1 (MRN) complex senses DSBs and stably recruits ATM [68,69], which can phosphorylate itself and downstream cellular targets, including MDC1. Then, RNF8 recognizes MDC1 and promotes the ubiquitylation of histone H1 [70,71]. RNF168 recognizes ubiquitylated H1 and recruits BRCA1 and 53BP1 to mediate the HR and NHEJ pathways, respectively [72,73]. In the next step, CtIP, Exo1, and BRCA1 are implicated in the DNA end resection. The emergent ssDNA protected by replication protein A (RPA), which BRCA2 displaces, invades duplex DNA molecules through the assistance of RAD51 and BRCA1–BARD1–PALB2. With sister chromatid DNA as a template, DNA Pol δ/ε chiefly mediates the nascent strand synthesis [74,75,76], while the SSA pathway directly joins two homologous 3′ ssDNA ends after extensive DNA end resection and RPA displacement, requiring RAD52, XPF–ERCC1 and LIG1 [77,78,79].

NHEJ does not require template DNA for repair, which distinguishes it from HR. It is an error-prone means of repair which can operate throughout the cell cycle. The Ku heterodimer (Ku70 and Ku80 subunits) is needed to recognize DSB termini [80]. Then, DNA-PK is recruited by binding Ku80 [81,82]. Finally, the XRCC4-XLF, Pol μ, and LIG4 complex joins the DNA ends together to complete the damage repair [83]. When the key NHEJ components are lacking, the A-EJ pathway, also known as microhomology-mediated end joining, is enhanced in the DDR [80,84]. It requires PARP1 and Pol θ (encoded by POLQ) to elicit the re-joining of the two DNA ends by using very short homologous sequences (2–20 bp). Due to the synthetic lethal relationship between HR and the A-EJ pathway, Pol θ is a novel druggable target for cancer therapy [85,86,87].

## 3. The Interplay between DDR Deficiency and Immune Response

**Figure 2:**
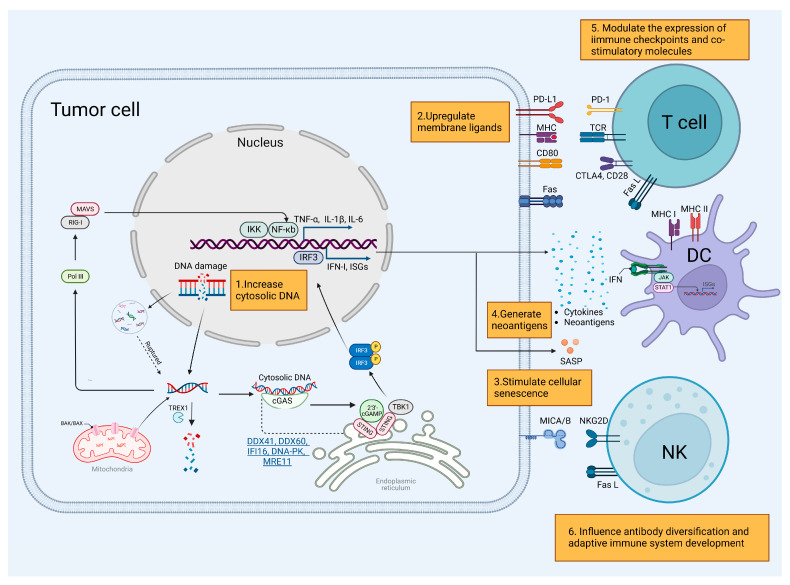
The interaction between DNA damage response (DDR) deficiency and immune response. The cytosolic DNA fragments, derived from nuclear, mitochondrial, or even extracellular DNA, are detected by sensors, including cGAS, DDX41, DDX60, IFNγ-inducible protein 16 (IFI16), DNA-PK, MRE11, and RNA polymerase III (Pol III). The cGAS-STING-IFN pathway plays an important role in innate immune response, which activates interferon regulatory factor 3 (IRF3) and nuclear factor κB (NF-κB) to produce IFN, ISG, and other cytokines, such as tumor necrosis factor (TNF)-α, interleukin (IL)-1β and IL-6. In other pathways, RNA polymerase III synthesizes 5′-PPP RNA from the AT-rich dsDNA or RNA: DNA hybrid to induce IFN-β through the RIG-I (retinoic acid-induced gene I)- MAVS (mitochondrial antiviral signaling) pathway. The produced IFNs activate JAK/STAT signaling pathway on the dendritic cells (DCs) to produce ISGs and proinflammatory cytokines and it also regulates many innate and adaptive immune cells, such as natural killer (NK) cells, CD8+ cytotoxic and CD4+ helper T cells. DDR deficiency also increases neoantigen and senescence-associated secretory phenotype (SASP) production. DDR process dysfunction can regulate ligand expression, including programmed cell death ligand 1 (PD-L1), Fas, CD80, and major histocompatibility complex class I-related molecules A and B (MICA and MICB) to alert the innate and adaptive immune systems. The potential molecular mechanisms of combining DDR target and immunotherapy are shown in the text box: (1) DDR deficiency increases cytosolic DNA generation, which elicits an innate immune response; (2) DDR activation prevents tumor cells from evading immunosurveillance by NK cells and/or CD8(+) T cells by shedding membrane ligands; (3) DNA damage triggers cellular senescence and promotes chronic inflammation; (4) DDR deficiency increases the number of neoantigens of the tumor, which enables a higher probability of recognition by the immune system; (5) DDR modulates the expression of immune checkpoints and other co-stimulatory molecules to lead immune escape; (6) DDR is critical for antibody diversification and influences the development of the adaptive immune system.

Figure 2 depicts the complex interaction between DDR deficiency and immune response.

### 3.1. Innate Immune Response

Genome instability is the hallmark of all forms of cancer [88], providing opportunities for intervention due to weak genome maintenance. DDR deficiency enhances genetic instability and imperfections [89], thus increasing endogenous nucleus-derived DNA generation in the cancer cell cytoplasm, which elicits an innate immune response.

#### 3.1.1. Cytosolic DNA Generation

The formation of cytosolic DNA includes cytosolic nucleosome-free DNA fragments, cytosolic chromatin fragments (CCF), and micronuclei (MN) [90,91,92], derived from nuclear DNA, mitochondrial DNA, or even extracellular nucleosomes as a result of DNA damage [93,94,95]. However, the molecular mechanisms of cytosolic DNA accumulation are still under exploration.

Defects in the DDR pathway cause replication forks to stall or collapse, leading to loss of chromosomal integrity maintenance and generating DNA fragments. For instance, a defect in MLH1 in the MMR system leads to a loss of regulation of Exo1. This causes unrestrained DNA end resection, leading to increased formation of ssDNA. Ultimately, this leads to chromosomal abnormalities and the release of nuclear DNA into the cytoplasm [34,96]. Similarly, MRE11 excessively degrades unprotected newly replicated genomes following RAD51 or BRCA2 dysfunction, resulting in increased fragmentation of nascent DNA [75,76,97,98]. On the other hand, the depletion of SAMHD1, which promotes the degradation of nascent DNA by stimulating the exonuclease activity of MRE11, leads to the release of ssDNA fragments [99]. This suggests a double-edged sword characteristic. In PARP-dependent DNA repair pathways, DNA structure-specific endonuclease MUS81 (a member of the XPF family) cleaves aberrant DNA structures at sites of stalled replication forks to preserve genome integrity [100,101].

Replication stress and unrepaired dsDNA also contribute to chromosomal instability [102,103]. Deletion of the interferon-stimulated gene (ISG15), which plays critical roles in the DDR to modulate p53 signaling and error-free DNA replication, was associated with CCF formation [104,105]. Interestingly, BLM RecQ-like helicase limits ISG induction to prevent genome instability [106]. Meanwhile, chromosomal instability leads to a preponderance of MN [107,108], which also results in the persistence of unrepaired DSBs during mitosis [91].

Mitochondrial DNA is also part of cytosolic DNA. Activation of intrinsic BAK and BAX–mediated apoptosis leads to the appearance of the BAK/BAX macropores, which allow the inner mitochondrial membrane to herniate into the cytosol, carrying matrix components, including the mtDNA [109]. Aberrant mtDNA packaging can also promote its escape into the cytosol, such as the loss of function of TFAM, an mtDNA packaging protein, which can elicit moderate mtDNA stress [93]. It is generally accepted that mtDNA activates DNA sensors upon its release into the cytoplasm [93,110].

To prevent host cytosolic DNA from accumulating and being recognized by DNA sensors, DNases degrade DNA molecules to maintain homeostatic conditions. For instance, DNase II rapidly degrades DNA derived from pathogens or apoptotic cells within endolysosomes [111], and three prime repair exonuclease (Trex1), a major DNA-specific 3′-5′ exonuclease in mammalian cells, degrades endogenous retroviruses and byproducts of DNA replication [112].

#### 3.1.2. Cytosolic Nucleic Acid Sensing Pathway

Pattern recognition receptors (PRRs), which include pathogen-associated molecular patterns (PAMPs) and damage-associated molecular patterns (DAMPs), detect cytosolic DNA and trigger innate immunity. When pathogenic nucleic acids are detected, the DNA sensor transduces a signal to the nucleus to produce proinflammatory cytokines. Among the downstream signaling for innate immune response, the cGAS-STING-IFN (cyclic GMP-AMP synthase, stimulator of interferon genes, interferon) pathway has been demonstrated to play an important role [113,114,115,116,117,118]. Defects in SWI/SNF subunits, including PBRM1, ARID1A, and SMARCA4, lead to replication stress and accumulation of cytosolic DNA, which facilitates cGAS–STING pathway activation following DNA damage [119,120,121].

Recognition of ruptured micronuclei or chromatin fragments by cGAS links genome instability to the innate immune response [3,122]. Upon the binding of cytosolic DNA, cGAS, as one of the most significant PRRs, catalyzes the synthesis of cyclic-dinucleotide 2′3′-cGAMP (cGAMP), which binds to STING at which point STING translocates from the endoplasmic reticulum to the Golgi apparatus, activating a variety of downstream signaling molecules [117,123]. It recruits and activates tank-binding kinase 1 (TBK1), which in turn phosphorylates STING to activate the interferon regulatory factor 3 (IRF3). Then, IRF3 translocases to the nucleus to induce ISGs and type 1 IFN (IFN-I) expression [124,125]. In parallel, it also activates IKK, which triggers the nuclear factor κB (NF-κB) signaling pathway to produce IFN-I, ISG, and proinflammatory cytokines, such as tumor necrosis factor (TNF)-α, interleukin (IL)-1β and IL-6 [107,123,126,127,128]. Considerable evidence now suggests that IFN-induced immune responses are crucial for cancer immunotherapy. The produced IFNs, binding to the heterodimer type I IFN receptors (IFNAR1/IFNAR2), activate the JAK/STAT signaling pathway on the dendritic cells (DCs) to produce ISGs and proinflammatory cytokines, such as IFNγ and IP-10 (CXCL10), which influence adaptive immunity. IFNs also regulate the maturation, migration, and activation of various innate and adaptive immune cells, such as natural killer (NK) cells, macrophages, plasma B cells, CD8+ cytotoxic, and CD4+ helper T cells [129].

Moreover, cGAS also localizes to the nucleus, where it plays a role in regulating the DDR. When in the nucleus, cGAS is recruited to dsDNA and interacts with PARP1 to suppress HR progression [130,131]. It also acts as a decelerator of DNA replication forks to suppress replication-associated DNA damage [132]. However, nucleosomes have a higher binding affinity for cGAS than dsDNA, but they have significantly lower potency for activating cGAS [94]. The above suggests a complex connection between cytoplasmic and nuclear functions of cGAS in DDR-immunity interplay.

There are several other cytosolic DNA sensors that regulate type I IFNs and cytokine production, including DDX41 [133,134], DDX60, IFNγ-inducible protein 16 (IFI16) [135], DNA-PK, and MRE11, which converge on STING [136]. Meanwhile, DNA-dependent activators of IFN-regulatory factors (DAI) directly trigger TBK1 activation [137]. In addition, absent in melanoma (AIM2)-like receptors, sensing dsDNA activates the ASC/Caspase1 inflammasome pathway to produce IL-1b instead [138]. There are also other pathways in which RNA polymerase III synthesizes 5′-PPP RNA from the AT-rich dsDNA or RNA: DNA hybrid, which induces IFN-β through the RIG-I (retinoic acid-induced gene I)- MAVS (mitochondrial antiviral signaling) pathway [139,140].

#### 3.1.3. Other Mechanisms

Furthermore, DDR activation prevents tumor cells from evading immunosurveillance of NK cells and/or CD8(+) T cells by shedding membrane ligands (through poorly understood mechanisms). Stimulation of ATR or ATM—major DNA damage checkpoints—can upregulate the ligands, which activate NKG2D receptors to alert the innate immune system [141]. Likewise, inhibition of DNA damage pathway components can also prevent the upregulation of major histocompatibility complex class I-related molecules A and B (MICA and MICB), which serve as membrane ligands [142,143].

Cellular senescence could be triggered by DNA damage, causing mammalian cells to enter an irreversible growth arrest that prevents abnormal cells from proliferating. This process is dependent on DDR regulators such as ATM/ATR, as well as the p53/p16 (INK4a) dependent pathway [144,145]. One key feature of senescence is the senescence-associated secretory phenotype (SASP), which involves the expression and secretion of various proinflammatory cytokines and chemokines. These secreted factors can stimulate the immune system and promote chronic inflammation either directly or indirectly, offering potential therapeutic opportunities [146,147,148].

### 3.2. Adaptive Immune Response

#### 3.2.1. Influence Tumor Antigenicity

Defects in DNA repair may increase the number of neoantigens in the tumor. For instance, low BER/SSBR gene expression leads to high neoantigen production, which enables a higher probability of recognition by the immune system [149,150]. The number of neoantigens is directly proportional to the number of non-synonymous mutations, which would be increased by a deficiency of multiple DNA repair pathways, including MMR, POLE/POLD1 (encoding the catalytic and proofreading subunits of Pol ε and Pol δ), and HR [151,152,153,154]. Studies have shown that neoantigen-reactive T cells [112,113] may be a key factor in the effectiveness of immunotherapy, particularly in tumors with a high tumor mutational burden (TMB). Cancer-associated antigens, including neoantigens derived from genetic alterations, are presented to CD8+ T cells through the major histocompatibility complex (MHC) on DCs, and professional antigen-presenting cells (APCs). However, most neoantigens are usually not recognized by the immune system, so identifying highly tumor-specific antigens is crucial for the development of personalized immunotherapy [155,156]. Recent technological advances allow new strategies to emerge in predicting, identifying, and validating neoantigens, with the ultimate goal of creating personalized vaccines for cancer treatment [157,158,159].

Additionally, tumors with high TMB resulting from dysfunction in the DDR process may have better clinical outcomes when treated with ICBs such as CTLA4 and PD1 in certain types of cancer [160]. This has been retrospectively validated in patients with advanced lung cancer, gastrointestinal carcinomas, ovarian cancer, skin melanomas, and glioma [159,161,162,163,164,165], suggesting that increased neoantigen burden is a predictive factor for a better outcome when using ICBs [115].

On the other hand, tumor aneuploidy, which is derived from chromosome instability, also provides an independent prognostic value as a biomarker [166,167]. A higher aneuploidy score is associated with poor prognosis among patients with lower-TMB (<80th percentile) tumors treated with immunotherapy [167] and non-small cell lung cancer (NSCLC) treated with radiotherapy and ICB [168]. Now, aneuploidy has been determined to affect immune cell action against the tumor adversely. However, the mechanisms underlying this observation are not well understood, with one proposed explanation being that most tumors with extensive aneuploidy often have fewer infiltrating immune cells [169,170].

#### 3.2.2. Immune Checkpoint Interaction

DDR defects have been shown to modulate the expression of immune checkpoints and other co-stimulatory molecules. PDL1 is one of the hot spots for immune checkpoint blockade, with links to DDR defects. Specifically, tumoral PDL1 expression is more common in dMMR cancers relative to MMR-intact tumors, which have been identified in colorectal and endometrial carcinomas [171,172,173]. Nevertheless, the loss of MMR proteins seems to be less correlated with tumoral PDL1 expression in breast carcinoma, where MMR gene mutations are less common [174]. PDL1 is primarily induced by IFNγ [175] through the JAK1/JAK2-STAT1/STAT2/STAT3-IRF1 axis [176]. This pathway is activated by innate immunity in response to damaged DNA [150]. PARP inhibitors (PARPi) have been shown to potentiate IFN-γ-induced PDL1 expression in NSCLC cell lines and pancreatic cancer [177,178]. PDL1 upregulation, mediated by DNA damage signaling [179], has been linked to ATM/ATR-CHK1 pathway activation in BER- or BRCA2-depleted cells, for example [150,180,181], or the cGAS-STING-TBK1-IRF3 pathway [182,183]. Furthermore, the greater release of DAMPs from excessive DNA damage promoted by DDR deficiency could also upregulate PDL1 expression in the neighboring surviving tumor cells, due to the TLR4/MyD88/TRIF signaling mediated by HMGB1 [184,185]. Expression of PDL1 in tumors can serve as a potent mechanism for potentially immunogenic tumors to escape from host immune responses by negatively regulating T-cell antigen receptor signaling by binding PD1 [8,175,186,187,188,189]. Finally, blockading PDL1-PD1 binding may result in the remission of advanced-stage cancer, although it does not necessarily mean that PDL1+ tumors have higher response rates [8]. On the other hand, intracellular PDL1 can protect the mRNA of NBS1, BRCA1, and other DNA damage-related genes from degradation, thereby increasing cellular resistance to DNA damage [190].

Moreover, the expression of a co-stimulatory molecule related to DDR has implications for the immune system, as it is required to activate CD8+ T cells [191]. Co-stimulatory B7-1/B7-2 signals on antigen-presenting cells, which interact with CD28 molecules on the T-cell surface, may induce clonal expansion and activation of cytotoxic T cells (CTLs). Increasing CD8(+) CD28(−) T-cell apoptosis compared to CD8(+) CD28(+) T cells is correlated with an impaired DDR following treatment with etoposide, a topoisomerase II inhibitor [192]. Similarly, CTLA4 can exacerbate the DDR and induces T-cell apoptosis [193].

#### 3.2.3. Induce Immunogenic Cell Death

Fas ligand (FasL/CD95L), triggering apoptotic cell death following ligation to Fas (CD95/APO-1), helps to maintain tumor cells in a state of immune privilege by inducing apoptosis of anti-tumor immune effector cells [194]. Therefore, FasL in tumor cells may decrease lymphocyte infiltration, reduce anti-tumor immunity in vivo and promote tumor development [195,196]. Conversely, Fas expression in various human cancer cells enhances the anti-tumor efficiency of CD8+ T or NK cells. In human colon cancer cohorts, Fas expression has been strongly correlated with dMMR and MSI-high (MSI-H) tumors, and it also induced senescence caused by chronic DNA damage [197].

#### 3.2.4. Role in Immunogenic Diversity

DDR kinases activated by purposeful genotoxic insults can regulate cell type-specific processes: variable gene segment recombination (VDJ), class-switch recombination (CSR), and somatic hypermutation (SHM) [198]. These processes are required for the normal development and function of immune responses [199], in which programmed DNA damage occurs at a specific site [200,201]. Multiple components of the DDR pathway are involved with these intermediates. For instance, DNA-PK, XLF4, SHLD1, and LIG4 participate in RAG-induced (in VDJ) or AID-initiated (in CSR) DSBs repair [202,203,204]. During SHM, error-prone non-canonical BER and/or MMR help to diversify mutations in the variable region of immunoglobulin genes to create high-affinity antibodies [205,206]. DNA repair is critical for antibody diversification and influences the development of the adaptive immune system [207,208]. Disturbances in the balance between enzymatic mutagenesis and DNA repair are at the basis of lymphoid malignancies [209,210]. This raises the intriguing possibility that therapeutic agents that target DDR proteins may be used to manipulate immune responses.

## 4. Combining DDR Inhibition and Immunotherapy

### 4.1. Potential Mechanism and Clinical Implication

Tumor immunotherapy, including ICB and adoptive cell transfer, can manipulate specific components of the immune system to reverse immunity suppression and target various cancers. PD1/PDL1 inhibitors and CTLA4 inhibitors have shown encouraging therapeutic effects in these approaches [211,212]. Nevertheless, only a minority of cancer patients respond to ICB in the clinic. Even among dMMR/MSI-H mCRC (metastatic colorectal cancer) patients for whom PD1 blockade is a guideline-recommended, first-line treatment option, response rates range between 30% and 50% [213,214]. These data suggest the existence of intrinsic resistance mechanisms, which are often contingent on the tumor microenvironment (TME) [215]. As a consequence, the development of novel therapeutic designs, as well as the discovery of biomarkers, are currently areas of intense research activity [216,217]. Combination regimens of traditional DNA-damaging approaches, such as chemotherapy drugs and radiotherapy, have been shown to enhance immunity by increasing antigens to stimulate T-cell-mediated immunity and modulating certain aspects of the immunosuppressive milieu [218,219]. Moreover, there is evidence to suggest that lower DDR factor expression in tumors may be associated with a better response to anticancer immunity, implying substantial potential benefits from DNA repair inhibitors [220]. Thus, there is considerable interest in combining ICB with DDR inhibition (DDRi), in order to enhance genomic instability and immunotherapy activity and potentially achieve additional anti-tumor responses [8] (Figure 2).

### 4.2. Treatment Strategies for Combining DDR Targets

DDR kinase inhibitors, such as those targeting PARP, ATM, ATR, DNA-PK, CHK1/2, BER, and WEE1, have been tested in clinical trials as a way to kill tumor cells, as cancer cells are more sensitive to compromised repair systems compared to normal cells (Table 1) [89,221]. With the expectation that the combination of DDRis with ICBs will show high potency, multiple studies exploring this combination are ongoing (Table 2). An archetypal example is PARPi, which have shown significant therapeutic efficacy in BRCA-deficient cancers by blocking BRCA-independent DNA repair in ovarian and breast cancer [222,223]. However, PARPi have only improved progression-free survival without reaching statistical significance in cancer-specific mortality in patients with germline BRCA mutations [224,225,226]. ICB has been proposed to optimize these clinical outcomes. In the BRCA1(−) tumor model, CTLA4 blockades combined with PARPi induce protective anti-tumor immunity and significant survival benefit by locally inducing anti-tumor immunity and increasing levels of IFNγ [227]. Accumulating evidence has also suggested that olaparib, a type of PARPi, triggers robust local and systemic anti-tumor immunity through a STING-dependent anti-tumor immune response independent of BRCA deficiency. This response can be further augmented by combining olaparib with PD1 blockade [228,229,230]. The clinical results of combining PARPi with an ICB, such as in advanced triple-negative breast cancer and advanced or metastatic non-small cell lung cancer [231,232], support further research on using this strategy in various cancers. Moreover, in PARP inhibitor-resistant cancers, PARG inhibitors may impair cancer cell survival by suppressing replication fork progression and show comparable killing ability [233,234]. This offers the potential for combining PARG inhibitors with ICB.

Although other DDRis are being evaluated as monotherapies or in combination with cytotoxic or molecularly targeted agents in solid tumors, only a few early-phase trials currently focus on combining them with ICB [221]. Clinical trials with AZD6738 [235], an ATR inhibitor, and AZD1775 (NCT02617277), a WEE1 inhibitor, individually as well as in combination with durvalumab in patients with advanced cancers, are currently ongoing. New DDRis are being developed, such as WRN inhibitors, which have shown promising synthetic lethal interaction with MSI tumors [236]. As dMMR cancers are exceptionally responsive to ICB [237,238], the viability of WRN inhibition plus ICB deserves further exploration.

### 4.3. DDR-Related Biomarkers for Predicting Immune Response

As our understanding of the relationship between DDR and immune responses continues to grow, it is expected that additional DDR-related biomarkers will be identified to predict a patient’s response more accurately to immunotherapy.

Several clinical trials have demonstrated that dMMR/MSI-H is significantly associated with long-term responses to immunotherapy and better prognosis in colorectal and non-colorectal malignancies treated with ICBs. Compared to chemotherapy, pembrolizumab has fewer treatment-related adverse events without compromising overall survival, supporting it as an efficacious first-line therapy [239,240]. In practice, pembrolizumab (anti-PD1) has been approved for dMMR/MSI-H refractory or metastatic solid tumors, and nivolumab (anti-PD1) for dMMR/MSI-H CRC [241,242,243]. One plausible hypothesis is that dMMR contributes to high TMB, though the specific mechanisms remain unclear [161]. TMB also has emerged as a promising biomarker of immunotherapy response across multiple cancer types. A high TMB may be a biomarker for identifying patients who will benefit from ICBs, irrespective of PDL1 expression level [244,245,246]. In many cases, TMB is a more reliable predictive marker for PD1 and PDL1 blockade immunotherapy response than PD1 or PDL1 expression; for example, the presence of ten or more mut/Mb was associated with improved response and prolonged progression-free survival, irrespective of tumor PDL1 expression in NSCLC [247,248].

Though higher TMB has been reported frequently in tumors with deleterious DDR gene alterations, mutations in different types of DDR pathways do not always exhibit high mutational load. In addition, clinical outcomes among patients with low TMB tumors are heterogeneous, with TMB status showing no ability to predict ICB-response in melanoma patients [162]. Recently, DDR scores quantifying the tumor signature of DDR pathways in tumors have provided new insights for guiding immunotherapeutic strategies. This is because DDR scores are not just closely associated with TMB and genome alteration, but also provide information regarding real-time DNA repair function [249]. There is evidence that patients with low DDR pathway signature scores might not benefit from a monoclonal anti-PD1 therapy, making these scores potentially useful for predicting treatment response in tumor tissues [163,164]. Similarly, studies have found that patients with high DDR scores have significantly higher survival rates after receiving ICBs compared to those with low DDR scores, while the reverse is true for traditional treatments [250]. Furthermore, tumor aneuploidy has been found to predict prognosis independently among patients with lower TMB (<80th percentile) tumors treated with immunotherapy [166,167].

## 5. Conclusions and Future Prospects

It has been reported that DDRi can enhance immune signaling within the TME and complement neoantigens. However, different forms of DDR defects may have varying effects on tumor immunogenicity. DDRi may not generate sufficient neoantigens in tumors with low neoantigen burden to stimulate an immune response. Meanwhile, it can also be challenging to reduce the immune-suppressive effects of DDRi. For example, PARPis and ICB have not produced dramatic responses in patients with BRCA1mut- and HR-deficient high-grade serous ovarian cancer, as PARPis can mediate immune resistance and tumor progression by upregulating VEGF-A. This has led to the development of combination therapies using PARPis, ICB, and bevacizumab (anti-VEGF) [251]. Combining DDRi with ICB, radiotherapy, or chemotherapy may also be a promising means for achieving a favorable balance between immunogenicity and TME. DNA-PK inhibitors are being studied in combination with radiation and ICB in clinical trials (NCT04068194, NCT03724890).

It is important to pay careful attention to specific therapeutic approaches for combination treatments. Optimizing the dose and schedule of DDRi agents may allow for increased tumor damage while sparing normal tissue by taking advantage of the differences in DDR and immune response between cancer and normal cells. The order in which combination drugs are administered and the line of therapy should also be considered. It is important to consider the toxicities of combination treatments versus monotherapy, as these can limit the development of combination therapies. Some DDR members are broad-spectrum and are necessary for maintaining homeostasis in normal tissues, which means that severe adverse events may occur when combined with ICBs. This is also the reason why many DDRis are eliminated in preclinical or phase I clinical trials.

To optimize the use of combination therapies involving DDRis and ICBs, more specific and sensitive biomarkers are needed to identify the most suitable patient population and predict treatment outcomes. DDR scores are likely to be important predictive factors, but the definition of DDR deficiency genes varies across different tumor types. It is controversial as to which mutated genes (distinguished as heterozygous or homozygous, germline or somatic) should be used to characterize DDR status in tumors. Under conditions of active anti-tumor immunity, DDR scores have been found to positively correlate with immune-related biomarkers, such as the number of T cells (such as CD4+ activated memory cells, CD8+ cells), T-cell receptor repertoire, PDL1 expression, and broad immune infiltrate. Thus, integrating immune biomarkers into the DDR score may improve its predictive ability.

In this review, we discuss the classical mechanism of DDR and its interplay with the immune system. We also present a compilation of studies on the combination of DDRi and ICBs for various cancer types, with the goal of inspiring new ideas for improving the efficacy of anti-tumor therapies and sparking innovation. While combination therapy has achieved impressive results in the clinic, increasing the success rate of treatment remains a challenge, and the rate of failure is still relatively high. A deeper understanding of the role of DDR in the immune system will be crucial for the design of future clinical trials.

## Figures and Tables

**Table 1:** DDR inhibitors in clinical trials.

Target	Drug	Tumor Type
PARP	Olaparib, Niraparib, Veliparib, Rucaparib	TNBC, SCLC, NSCLC, ovarian cancer, bladder cancer, prostate, colorectal cancers, pancreatic cancer, advanced solid tumors
ATM	AZD-1390, M-4076, XRD-0394, AZD0156, M3541	Solid tumors
ATR	Berzosertib, Ceralasertib, RP-3500, ART-0380, ATRN-119, M-4344, M-1774, Elimusertib	Ovarian, Advanced NSCLC, SCLC, Gynae or neuroendocrine, HNSCC, relapsed CLL, PLL, B-cell lymphomas
DNA-PK	M3814, AZD-7648, CC-115, BR2002, BR101801	GBM, HNSCC, prostate, ES, CLL
MEK1/2	Selumetinib, Binimetinib, Cobinetinib, Trametinib	Melanoma, colorectal cancer, NF1-associated neurofibroma
WEE1	Adavosertib, ZN-c3, IMP7068, SY-4835, Debio0123	Endometrial serous carcinoma, osteosarcoma, solid tumor, NSCLC, gastric carcinoma, AML, other myeloid malignancies
CHK1/2	MK8776, LY2603618, CCT245737, LY2606368	NSCLC, refractory SCLC, relapsed AML, relapsed lymphoma, pancreatic carcinoma, ovarian, breast, prostate, pediatric solid tumors
BER	TRC102	GBM, lymphoma, hematologic malignancies, NSCLC

Abbreviation: AML, acute myeloid leukemia; CLL, chronic lymphocytic leukemia; DDRi, DNA Damage Response inhibition; DLT, dose-limiting toxicity; ES, Ewing sarcoma; GBM, glioblastoma multiforme; HNSCC, head, and neck squamous cell carcinoma; ICB, immune checkpoint blockade; NSCLC, non–small cell lung carcinoma; ORR, overall response rate; OS, overall survival; PFS, progression-free survival; PLL, prolymphocytic leukemia; SCLC, small cell lung carcinoma; TNBC, triple-negative breast cancer.

**Table 2:** Clinical Trials Using immune checkpoint blockade (ICB) + DNA Damage Response inhibition (DDRi). (https://clinicaltrials.gov (accessed on 1 November 2022)).

ICBs	DDRi	Study Identifier (Status)	Tumor Type	Phase	Primary Endpoint
Pembrolizumab	Niraparib	NCT02657889 (completed)	Ovarian, advanced TNBC	I/II	DLTs, ORR
		NCT04475939 (recruiting)	Advanced or metastatic NSCLC	III	3-year PFS, 5-year OS
		NCT03308942 (completed)	NSCLC	II	ORR
	Olaparib	NCT04191135 (not recruiting)	TNBC	II/III	PFS
		NCT04548752 (recruiting)	Inherited BRCA-mutation pancreatic cancer	II	PFS
		NCT02861573 (recruiting)	Metastatic castration-resistant prostate cancer	Ib/II	ORR
Nivolumab	Rucaparib	NCT03338790 (not recruiting)	Metastatic castration-resistant prostate cancer	I	ORR
		NCT03522246 (not recruiting)	Ovarian cancer	III	7-year PFS
		NCT03572478 (terminated)	Prostate or endometrial cancer	Ib/II	DLTs
	Veliparib	NCT02944396 (completed)	Metastatic or advanced NSCLC	II	PFS
Camrelizumab (SHR-1210)	Apatinib	NCT03394287 (completed)	Advanced TNBC	II	ORR
Tislelizumab (BGB-A317)	Pamiparib (BGB-290)	NCT02660034 (completed)	Advanced solid tumors	I	AEs
Dostarlimab (TSR-042)	Niraparib	NCT03307785 (not recruiting)	Solid tumor	I/II	DLTs
		NCT03602859 (not recruiting)	III or IV nonmucinous epithelial ovarian cancer	III	5-year PFS
Durvalumab	Olaparib	NCT03167619 (completed)	Advanced TNBC	II	1-year PFS
		NCT02734004 (not recruiting)	Ovarian, breast, SCLC, gastric cancers	I/II	Disease control rate
		NCT02546661 (not recruiting)	Muscle-invasive bladder cancer	I	AEs
		NCT03459846 (not recruiting)	Urinary bladder neoplasms	II	PFS
		NCT02484404 (recruiting)	Recurrent ovarian cancer	I/II	ORR
		NCT03334617 (recruiting)	Advanced NSCLC	II	ORR
Durvalumab	Olaparib	NCT03534492 (completed)	Resectable urothelial bladder cancer	II	Pathological complete response rate
Durvalumab+ Tremelimumab	Olaparib	NCT02953457 (not recruiting)	Recurrent or refractory ovarian, fallopian tube, or primary peritoneal cancer with BRCA1 or BRCA2 mutation	II	DLTs
Durvalumab	Olaparib	NCT03851614 (not recruiting)	Mismatch repair proficient colorectal cancer, pancreatic adenocarcinoma, leiomyosarcoma	II	Changes in genomic and immune biomarkers
	Ceralasertib	NCT02264678 (recruiting)	Advanced Solid Tumors	I/II	AEs
	Olaparib	NCT02484404 (recruiting)	Advanced Solid Tumors and Advanced or Recurrent Ovarian, TN BC, Lung, Prostate, and Colorectal Cancers	I/II	ORR
	Adavosertib (AZD1775)	NCT02617277 (not recruiting)	Advanced solid tumors	I	DLTs
Ipilimumab or Nivolumab	Niraparib	NCT03404960 (not recruiting)	Pancreatic adenocarcinoma	I/II	PFS
Avelumab	Talazoparib	NCT03330405 (not recruiting)	Locally advanced (primary or recurrent) or metastatic solid tumors	Ib/II	DLTs
		NCT03565991. (not recruiting)	BRCA1/2 or ATM alterations tumor	II	Confirmed Objective Response
		NCT03637491 (terminated)	Locally advanced or metastatic RAS-mutant solid tumors	Ib/II	DLTs
		NCT03642132 (completed)	Ovarian cancer	III	PFS
		NCT03964532 (not recruiting)	Advanced breast cancer	I/II	AEs
Atezolizumab	Olaparib	NCT02849496 (not recruiting)	Mutant Non-HER2-positive breast cancer	II	PFS
	Rucaparib	NCT03101280 (completed)	Advanced gynecologic cancers, TNBC	IB	AEs
	Niraparib	NCT03598270 (not recruiting)	Recurrent ovarian cancer	III	PFS
Tremelimumab	Olaparib	NCT02571725 (not recruiting)	BRCA-deficient ovarian cancer	I/II	DLT

Abbreviation: ICBs, immune checkpoint blocks; DDRi, DNA damage response inhibitors; DLTs, dose-limiting toxicities; ORR, overall response rate; TNBC, triple-negative breast cancer; NSCLC, non-small cell lung cancer; PFS, Progression-Free Survival; AEs, adverse events.

## References

[1] Siegel R.L., Miller K.D., Fuchs H.E., Jemal A. (2022). Cancer Statistics, 2022. CA. Cancer J. Clin..

[2] Pérez-Herrero E., Fernández-Medarde A. (2015). Advanced Targeted Therapies in Cancer: Drug Nanocarriers, the Future of Chemotherapy. Eur. J. Pharm. Biopharm..

[3] Hait W.N. (2009). Targeted Cancer Therapeutics. Cancer Res..

[4] Middleton G., Robbins H., Andre F., Swanton C. (2022). A State-of-the-Art Review of Stratified Medicine in Cancer: Towards a Future Precision Medicine Strategy in Cancer. Ann. Oncol..

[5] Zhu S., Zhang T., Zheng L., Liu H., Song W., Liu D., Li Z., Pan C. (2021). Combination Strategies to Maximize the Benefits of Cancer Immunotherapy. J. Hematol. Oncol..

[6] Zimmer L., Livingstone E., Hassel J.C., Fluck M., Eigentler T., Loquai C., Haferkamp S., Gutzmer R., Meier F., Mohr P. (2020). Adjuvant Nivolumab plus Ipilimumab or Nivolumab Monotherapy versus Placebo in Patients with Resected Stage IV Melanoma with No Evidence of Disease (IMMUNED): A Randomised, Double-Blind, Placebo-Controlled, Phase 2 Trial. Lancet.

[7] Robert C., Ribas A., Schachter J., Arance A., Grob J.-J., Mortier L., Daud A., Carlino M.S., McNeil C.M., Lotem M. (2019). Pembrolizumab versus Ipilimumab in Advanced Melanoma (KEYNOTE-006): Post-Hoc 5-Year Results from an Open-Label, Multicentre, Randomised, Controlled, Phase 3 Study. Lancet Oncol..

[8] Kim T.K., Vandsemb E.N., Herbst R.S., Chen L. (2022). Adaptive Immune Resistance at the Tumour Site: Mechanisms and Therapeutic Opportunities. Nat. Rev. Drug Discov..

[9] O’Connor M.J. (2015). Targeting the DNA Damage Response in Cancer. Mol. Cell.

[10] Dobbelstein M., Sørensen C.S. (2015). Exploiting Replicative Stress to Treat Cancer. Nat. Rev. Drug Discov..

[11] Chatzinikolaou G., Karakasilioti I., Garinis G.A. (2014). DNA Damage and Innate Immunity: Links and Trade-Offs. Trends Immunol..

[12] Mouw K.W., Goldberg M.S., Konstantinopoulos P.A., D’Andrea A.D. (2017). DNA Damage and Repair Biomarkers of Immunotherapy Response. Cancer Discov..

[13] Nastasi C., Mannarino L., D’Incalci M. (2020). DNA Damage Response and Immune Defense. Int. J. Mol. Sci..

[14] Galon J., Costes A., Sanchez-Cabo F., Kirilovsky A., Mlecnik B., Lagorce-Pagès C., Tosolini M., Camus M., Berger A., Wind P. (2006). Type, Density, and Location of Immune Cells within Human Colorectal Tumors Predict Clinical Outcome. Science.

[15] Dexheimer T.S., Mathews L.A., Cabarcas S.M., Hurt E.M. (2013). DNA Repair Pathways and Mechanisms. DNA Repair of Cancer Stem Cells.

[16] Hoeijmakers J.H.J. (2009). DNA Damage, Aging, and Cancer. N. Engl. J. Med..

[17] Sirbu B.M., Cortez D. (2013). DNA Damage Response: Three Levels of DNA Repair Regulation. Cold Spring Harb. Perspect. Biol..

[18] Curtin N.J. (2012). DNA Repair Dysregulation from Cancer Driver to Therapeutic Target. Nat. Rev. Cancer.

[19] Christmann M., Tomicic M.T., Roos W.P., Kaina B. (2003). Mechanisms of Human DNA Repair: An Update. Toxicology.

[20] Huang R., Zhou P.-K. (2021). DNA Damage Repair: Historical Perspectives, Mechanistic Pathways and Clinical Translation for Targeted Cancer Therapy. Signal Transduct. Target. Ther..

[21] Chatterjee N., Walker G.C. (2017). Mechanisms of DNA Damage, Repair, and Mutagenesis: DNA Damage and Repair. Environ. Mol. Mutagen..

[22] Zharkov D.O. (2008). Base Excision DNA Repair. Cell. Mol. Life Sci..

[23] Fortini P., Dogliotti E. (2007). Base Damage and Single-Strand Break Repair: Mechanisms and Functional Significance of Short- and Long-Patch Repair Subpathways. DNA Repair.

[24] Barakat K.H., Gajewski M.M., Tuszynski J.A. (2012). DNA Polymerase Beta (Pol β) Inhibitors: A Comprehensive Overview. Drug Discov. Today.

[25] Chang D.S., Lasley F.D., Das I.J., Mendonca M.S., Dynlacht J.R. (2014). Molecular Mechanisms of DNA Damage and Repair. Basic Radiotherapy Physics and Biology.

[26] Shuck S.C., Short E.A., Turchi J.J. (2008). Eukaryotic Nucleotide Excision Repair: From Understanding Mechanisms to Influencing Biology. Cell Res..

[27] Nouspikel T. (2008). Nucleotide Excision Repair and Neurological Diseases. DNA Repair.

[28] Hanawalt P.C., Spivak G. (2008). Transcription-Coupled DNA Repair: Two Decades of Progress and Surprises. Nat. Rev. Mol. Cell Biol..

[29] Sugasawa K. (2010). Regulation of Damage Recognition in Mammalian Global Genomic Nucleotide Excision Repair. Mutat. Res. Mol. Mech. Mutagen..

[30] Thoms K.-M., Kuschal C., Emmert S. (2007). Lessons Learned from DNA Repair Defective Syndromes. Exp. Dermatol..

[31] Poulogiannis G., Frayling I.M., Arends M.J. (2010). DNA Mismatch Repair Deficiency in Sporadic Colorectal Cancer and Lynch Syndrome. Histopathology.

[32] Ortega J., Lee G.S., Gu L., Yang W., Li G.-M. (2021). Mispair-Bound Human MutS–MutL Complex Triggers DNA Incisions and Activates Mismatch Repair. Cell Res..

[33] Tran P.T., Erdeniz N., Symington L.S., Liskay R.M. (2004). EXO1-A Multi-Tasking Eukaryotic Nuclease. DNA Repair.

[34] Guan J., Lu C., Jin Q., Lu H., Chen X., Tian L., Zhang Y., Ortega J., Zhang J., Siteni S. (2021). MLH1 Deficiency-Triggered DNA Hyperexcision by Exonuclease 1 Activates the CGAS-STING Pathway. Cancer Cell.

[35] Niraj J., Färkkilä A., D’Andrea A.D. (2019). The Fanconi Anemia Pathway in Cancer. Annu. Rev. Cancer Biol..

[36] Zhang J., Dewar J.M., Budzowska M., Motnenko A., Cohn M.A., Walter J.C. (2015). DNA Interstrand Cross-Link Repair Requires Replication-Fork Convergence. Nat. Struct. Mol. Biol..

[37] Rodríguez A., D’Andrea A. (2017). Fanconi Anemia Pathway. Curr. Biol..

[38] Shakeel S., Rajendra E., Alcón P., O’Reilly F., Chorev D.S., Maslen S., Degliesposti G., Russo C.J., He S., Hill C.H. (2019). Structure of the Fanconi Anaemia Monoubiquitin Ligase Complex. Nature.

[39] Tan W., van Twest S., Murphy V.J., Deans A.J. (2020). ATR-Mediated FANCI Phosphorylation Regulates Both Ubiquitination and Deubiquitination of FANCD2. Front. Cell Dev. Biol..

[40] Kolinjivadi A.M., Crismani W., Ngeow J. (2020). Emerging Functions of Fanconi Anemia Genes in Replication Fork Protection Pathways. Hum. Mol. Genet..

[41] Peake J.D., Noguchi E. (2022). Fanconi Anemia: Current Insights Regarding Epidemiology, Cancer, and DNA Repair. Hum. Genet..

[42] Amunugama R., Walter J.C. (2020). A New Varietal of DNA Interstrand Crosslink Repair. Cell Res..

[43] Yang W., Gao Y. (2018). Translesion and Repair DNA Polymerases: Diverse Structure and Mechanism. Annu. Rev. Biochem..

[44] Bezalel-Buch R., Cheun Y.K., Roy U., Schärer O.D., Burgers P.M. (2020). Bypass of DNA Interstrand Crosslinks by a Rev1–DNA Polymerase ζ Complex. Nucleic Acids Res..

[45] Budzowska M., Graham T.G., Sobeck A., Waga S., Walter J.C. (2015). Regulation of the Rev1–Pol ζ Complex during Bypass of a DNA Interstrand Cross-link. EMBO J..

[46] Shen S., Davidson G.A., Yang K., Zhuang Z. (2021). Photo-Activatable Ub-PCNA Probes Reveal New Structural Features of the *Saccharomyces Cerevisiae* Polη/PCNA Complex. Nucleic Acids Res..

[47] Weaver T.M., Click T.H., Khoang T.H., Todd Washington M., Agarwal P.K., Freudenthal B.D. (2022). Mechanism of Nucleotide Discrimination by the Translesion Synthesis Polymerase Rev1. Nat. Commun..

[48] Masłowska K.H., Villafañez F., Laureti L., Iwai S., Pagès V. (2022). Eukaryotic Stress–Induced Mutagenesis Is Limited by a Local Control of Translesion Synthesis. Nucleic Acids Res..

[49] Fujii S., Fuchs R.P. (2020). A Comprehensive View of Translesion Synthesis in Escherichia Coli. Microbiol. Mol. Biol. Rev..

[50] Lv L., Wang F., Ma X., Yang Y., Wang Z., Liu H., Li X., Liu Z., Zhang T., Huang M. (2013). Mismatch Repair Protein MSH2 Regulates Translesion DNA Synthesis Following Exposure of Cells to UV Radiation. Nucleic Acids Res..

[51] Paniagua I., Tayeh Z., Falcone M., Hernández Pérez S., Cerutti A., Jacobs J.J.L. (2022). MAD2L2 Promotes Replication Fork Protection and Recovery in a Shieldin-Independent and REV3L-Dependent Manner. Nat. Commun..

[52] Chen D., Gervai J.Z., Póti Á., Németh E., Szeltner Z., Szikriszt B., Gyüre Z., Zámborszky J., Ceccon M., d’Adda di Fagagna F. (2022). BRCA1 Deficiency Specific Base Substitution Mutagenesis Is Dependent on Translesion Synthesis and Regulated by 53BP1. Nat. Commun..

[53] Clairmont C.S., Sarangi P., Ponnienselvan K., Galli L.D., Csete I., Moreau L., Adelmant G., Chowdhury D., Marto J.A., D’Andrea A.D. (2020). TRIP13 Regulates DNA Repair Pathway Choice through REV7 Conformational Change. Nat. Cell Biol..

[54] Taglialatela A., Leuzzi G., Sannino V., Cuella-Martin R., Huang J.-W., Wu-Baer F., Baer R., Costanzo V., Ciccia A. (2021). REV1-Polζ Maintains the Viability of Homologous Recombination-Deficient Cancer Cells through Mutagenic Repair of PRIMPOL-Dependent SsDNA Gaps. Mol. Cell.

[55] Caldecott K.W. (2003). Protein–Protein Interactions during Mammalian DNA Single-Strand Break Repair. Biochem. Soc. Trans..

[56] Caldecott K.W. (2022). DNA Single-Strand Break Repair and Human Genetic Disease. Trends Cell Biol..

[57] Blair K., Tehseen M., Raducanu V.-S., Shahid T., Lancey C., Rashid F., Crehuet R., Hamdan S.M., De Biasio A. (2022). Mechanism of Human Lig1 Regulation by PCNA in Okazaki Fragment Sealing. Nat. Commun..

[58] Mengwasser K.E., Adeyemi R.O., Leng Y., Choi M.Y., Clairmont C., D’Andrea A.D., Elledge S.J. (2019). Genetic Screens Reveal FEN1 and APEX2 as BRCA2 Synthetic Lethal Targets. Mol. Cell.

[59] Williams J.S., Tumbale P.P., Arana M.E., Rana J.A., Williams R.S., Kunkel T.A. (2021). High-Fidelity DNA Ligation Enforces Accurate Okazaki Fragment Maturation during DNA Replication. Nat. Commun..

[60] Saha L.K., Wakasugi M., Akter S., Prasad R., Wilson S.H., Shimizu N., Sasanuma H., Huang S.N., Agama K., Pommier Y. (2020). Topoisomerase I-Driven Repair of UV-Induced Damage in NER-Deficient Cells. Proc. Natl. Acad. Sci. USA.

[61] Lin Y., Raj J., Li J., Ha A., Hossain M.A., Richardson C., Mukherjee P., Yan S. (2020). APE1 Senses DNA Single-Strand Breaks for Repair and Signaling. Nucleic Acids Res..

[62] Scully R., Panday A., Elango R., Willis N.A. (2019). DNA Double-Strand Break Repair-Pathway Choice in Somatic Mammalian Cells. Nat. Rev. Mol. Cell Biol..

[63] Cejka P., Symington L.S. (2021). DNA End Resection: Mechanism and Control. Annu. Rev. Genet..

[64] Xu Y., Xu D. (2020). Repair Pathway Choice for Double-Strand Breaks. Essays Biochem..

[65] Atkinson J., Bezak E., Kempson I. (2022). Imaging DNA Double-Strand Breaks—Are We There Yet?. Nat. Rev. Mol. Cell Biol..

[66] Pardo B., Gómez-González B., Aguilera A. (2009). DNA Repair in Mammalian Cells: DNA Double-Strand Break Repair: How to Fix a Broken Relationship. Cell. Mol. Life Sci..

[67] Li X., Heyer W.-D. (2008). Homologous Recombination in DNA Repair and DNA Damage Tolerance. Cell Res..

[68] Lee J.-H., Paull T.T. (2005). ATM Activation by DNA Double-Strand Breaks Through the Mre11-Rad50-Nbs1 Complex. Science.

[69] Abraham R.T., Tibbetts R.S. (2005). Guiding ATM to Broken DNA. Science.

[70] Mailand N., Bekker-Jensen S., Faustrup H., Melander F., Bartek J., Lukas C., Lukas J. (2007). RNF8 Ubiquitylates Histones at DNA Double-Strand Breaks and Promotes Assembly of Repair Proteins. Cell.

[71] Thorslund T., Ripplinger A., Hoffmann S., Wild T., Uckelmann M., Villumsen B., Narita T., Sixma T.K., Choudhary C., Bekker-Jensen S. (2015). Histone H1 Couples Initiation and Amplification of Ubiquitin Signalling after DNA Damage. Nature.

[72] Doil C., Mailand N., Bekker-Jensen S., Menard P., Larsen D.H., Pepperkok R., Ellenberg J., Panier S., Durocher D., Bartek J. (2009). RNF168 Binds and Amplifies Ubiquitin Conjugates on Damaged Chromosomes to Allow Accumulation of Repair Proteins. Cell.

[73] Hustedt N., Durocher D. (2017). The Control of DNA Repair by the Cell Cycle. Nat. Cell Biol..

[74] Prakash R., Zhang Y., Feng W., Jasin M. (2015). Homologous Recombination and Human Health: The Roles of BRCA1, BRCA2, and Associated Proteins. Cold Spring Harb. Perspect. Biol..

[75] Jensen R.B., Carreira A., Kowalczykowski S.C. (2010). Purified Human BRCA2 Stimulates RAD51-Mediated Recombination. Nature.

[76] Yang H., Li Q., Fan J., Holloman W.K., Pavletich N.P. (2005). The BRCA2 Homologue Brh2 Nucleates RAD51 Filament Formation at a DsDNA–SsDNA Junction. Nature.

[77] Benitez A., Liu W., Palovcak A., Wang G., Moon J., An K., Kim A., Zheng K., Zhang Y., Bai F. (2018). FANCA Promotes DNA Double-Strand Break Repair by Catalyzing Single-Strand Annealing and Strand Exchange. Mol. Cell.

[78] Mendez-Dorantes C., Bhargava R., Stark J.M. (2018). Repeat-Mediated Deletions Can Be Induced by a Chromosomal Break Far from a Repeat, but Multiple Pathways Suppress Such Rearrangements. Genes Dev..

[79] Bhargava R., Onyango D.O., Stark J.M. (2016). Regulation of Single-Strand Annealing and Its Role in Genome Maintenance. Trends Genet..

[80] Zhao B., Rothenberg E., Ramsden D.A., Lieber M.R. (2020). The Molecular Basis and Disease Relevance of Non-Homologous DNA End Joining. Nat. Rev. Mol. Cell Biol..

[81] Blackford A.N., Jackson S.P. (2017). ATM, ATR, and DNA-PK: The Trinity at the Heart of the DNA Damage Response. Mol. Cell.

[82] Gell D., Jackson S.P. (1999). Mapping of Protein-Protein Interactions within the DNA-Dependent Protein Kinase Complex. Nucleic Acids Res..

[83] Ahnesorg P., Smith P., Jackson S.P. (2006). XLF Interacts with the XRCC4-DNA Ligase IV Complex to Promote DNA Nonhomologous End-Joining. Cell.

[84] Zhou J., Gelot C., Pantelidou C., Li A., Yücel H., Davis R.E., Färkkilä A., Kochupurakkal B., Syed A., Shapiro G.I. (2021). A First-in-Class Polymerase Theta Inhibitor Selectively Targets Homologous-Recombination-Deficient Tumors. Nat. Cancer.

[85] Villanueva M.T. (2015). A New Tool to Target DNA Repair. Nat. Rev. Cancer.

[86] Helleday T. (2021). Polθ Inhibitors Unchained. Nat. Cancer.

[87] Ceccaldi R., Liu J.C., Amunugama R., Hajdu I., Primack B., Petalcorin M.I.R., O’Connor K.W., Konstantinopoulos P.A., Elledge S.J., Boulton S.J. (2015). Homologous-Recombination-Deficient Tumours Are Dependent on Polθ-Mediated Repair. Nature.

[88] Hanahan D. (2022). Hallmarks of Cancer: New Dimensions. Cancer Discov..

[89] Jackson S.P., Helleday T. (2016). Drugging DNA Repair. Science.

[90] Kwon J., Bakhoum S.F. (2020). The Cytosolic DNA-Sensing CGAS–STING Pathway in Cancer. Cancer Discov..

[91] Harding S.M., Benci J.L., Irianto J., Discher D.E., Minn A.J., Greenberg R.A. (2017). Mitotic Progression Following DNA Damage Enables Pattern Recognition within Micronuclei. Nature.

[92] Bakhoum S., Ngo B., Bakhoum A., Cavallo-Fleming J.A., Murphy C.W., Powell S.N., Cantley L. (2018). Chromosomal Instability Drives Metastasis Through a Cytosolic DNA Response. Int. J. Radiat. Oncol..

[93] West A.P., Khoury-Hanold W., Staron M., Tal M.C., Pineda C.M., Lang S.M., Bestwick M., Duguay B.A., Raimundo N., MacDuff D.A. (2015). Mitochondrial DNA Stress Primes the Antiviral Innate Immune Response. Nature.

[94] Wang H., Zang C., Ren M., Shang M., Wang Z., Peng X., Zhang Q., Wen X., Xi Z., Zhou C. (2020). Cellular Uptake of Extracellular Nucleosomes Induces Innate Immune Responses by Binding and Activating CGMP-AMP Synthase (CGAS). Sci. Rep..

[95] White M.J., McArthur K., Metcalf D., Lane R.M., Cambier J.C., Herold M.J., van Delft M.F., Bedoui S., Lessene G., Ritchie M.E. (2014). Apoptotic Caspases Suppress MtDNA-Induced STING-Mediated Type I IFN Production. Cell.

[96] Lu C., Guan J., Lu S., Jin Q., Rousseau B., Lu T., Stephens D., Zhang H., Zhu J., Yang M. (2021). DNA Sensing in Mismatch Repair-Deficient Tumor Cells Is Essential for Anti-Tumor Immunity. Cancer Cell.

[97] Bhattacharya S., Srinivasan K., Abdisalaam S., Su F., Raj P., Dozmorov I., Mishra R., Wakeland E.K., Ghose S., Mukherjee S. (2017). RAD51 Interconnects between DNA Replication, DNA Repair and Immunity. Nucleic Acids Res..

[98] Costanzo V. (2011). Brca2, Rad51 and Mre11: Performing Balancing Acts on Replication Forks. DNA Repair.

[99] Coquel F., Silva M.-J., Técher H., Zadorozhny K., Sharma S., Nieminuszczy J., Mettling C., Dardillac E., Barthe A., Schmitz A.-L. (2018). SAMHD1 Acts at Stalled Replication Forks to Prevent Interferon Induction. Nature.

[100] Ciccia A., McDonald N., West S.C. (2008). Structural and Functional Relationships of the XPF/MUS81 Family of Proteins. Annu. Rev. Biochem..

[101] Ho S.S.W., Zhang W.Y.L., Tan N.Y.J., Khatoo M., Suter M.A., Tripathi S., Cheung F.S.G., Lim W.K., Tan P.H., Ngeow J. (2016). The DNA Structure-Specific Endonuclease MUS81 Mediates DNA Sensor STING-Dependent Host Rejection of Prostate Cancer Cells. Immunity.

[102] Cybulla E., Vindigni A. (2023). Leveraging the Replication Stress Response to Optimize Cancer Therapy. Nat. Rev. Cancer.

[103] García-de-Teresa B., Rodríguez A., Frias S. (2020). Chromosome Instability in Fanconi Anemia: From Breaks to Phenotypic Consequences. Genes.

[104] Wardlaw C.P., Petrini J.H.J. (2022). ISG15 Conjugation to Proteins on Nascent DNA Mitigates DNA Replication Stress. Nat. Commun..

[105] Sandy Z., da Costa I.C., Schmidt C.K. (2020). More than Meets the ISG15: Emerging Roles in the DNA Damage Response and Beyond. Biomolecules.

[106] Gratia M., Rodero M.P., Conrad C., Bou Samra E., Maurin M., Rice G.I., Duffy D., Revy P., Petit F., Dale R.C. (2019). Bloom Syndrome Protein Restrains Innate Immune Sensing of Micronuclei by CGAS. J. Exp. Med..

[107] Hong C., Schubert M., Tijhuis A.E., Requesens M., Roorda M., van den Brink A., Ruiz L.A., Bakker P.L., van der Sluis T., Pieters W. (2022). CGAS–STING Drives the IL-6-Dependent Survival of Chromosomally Instable Cancers. Nature.

[108] Bakhoum S.F., Ngo B., Laughney A.M., Cavallo J.-A., Murphy C.J., Ly P., Shah P., Sriram R.K., Watkins T.B.K., Taunk N.K. (2018). Chromosomal Instability Drives Metastasis through a Cytosolic DNA Response. Nature.

[109] McArthur K., Whitehead L.W., Heddleston J.M., Li L., Padman B.S., Oorschot V., Geoghegan N.D., Chappaz S., Davidson S., San Chin H. (2018). BAK/BAX Macropores Facilitate Mitochondrial Herniation and MtDNA Efflux during Apoptosis. Science.

[110] Kanneganti T.-D., Kundu M., Green D.R. (2015). Innate Immune Recognition of MtDNA—An Undercover Signal?. Cell Metab..

[111] Chan M.P., Onji M., Fukui R., Kawane K., Shibata T., Saitoh S., Ohto U., Shimizu T., Barber G.N., Miyake K. (2015). DNase II-Dependent DNA Digestion Is Required for DNA Sensing by TLR9. Nat. Commun..

[112] Yang Y.-G., Lindahl T., Barnes D.E. (2007). Trex1 Exonuclease Degrades SsDNA to Prevent Chronic Checkpoint Activation and Autoimmune Disease. Cell.

[113] Ablasser A., Chen Z.J. (2019). CGAS in Action: Expanding Roles in Immunity and Inflammation. Science.

[114] Du H., Xu T., Cui M. (2021). CGAS-STING Signaling in Cancer Immunity and Immunotherapy. Biomed. Pharmacother..

[115] Hopfner K.-P., Hornung V. (2020). Molecular Mechanisms and Cellular Functions of CGAS–STING Signalling. Nat. Rev. Mol. Cell Biol..

[116] Wan D., Jiang W., Hao J. (2020). Research Advances in How the CGAS-STING Pathway Controls the Cellular Inflammatory Response. Front. Immunol..

[117] Burdette D.L., Monroe K.M., Sotelo-Troha K., Iwig J.S., Eckert B., Hyodo M., Hayakawa Y., Vance R.E. (2011). STING Is a Direct Innate Immune Sensor of Cyclic Di-GMP. Nature.

[118] Zhang X., Bai X., Chen Z.J. (2020). Structures and Mechanisms in the CGAS-STING Innate Immunity Pathway. Immunity.

[119] Ribeiro-Silva C., Aydin Ö.Z., Mesquita-Ribeiro R., Slyskova J., Helfricht A., Marteijn J.A., Hoeijmakers J.H.J., Lans H., Vermeulen W. (2018). DNA Damage Sensitivity of SWI/SNF-Deficient Cells Depends on TFIIH Subunit P62/GTF2H1. Nat. Commun..

[120] Wang L., Yang L., Wang C., Zhao W., Ju Z., Zhang W., Shen J., Peng Y., An C., Luu Y.T. (2020). Inhibition of the ATM/Chk2 Axis Promotes CGAS/STING Signaling in ARID1A-Deficient Tumors. J. Clin. Investig..

[121] Chabanon R.M., Morel D., Eychenne T., Colmet-Daage L., Bajrami I., Dorvault N., Garrido M., Meisenberg C., Lamb A., Ngo C. (2021). PBRM1 Deficiency Confers Synthetic Lethality to DNA Repair Inhibitors in Cancer. Cancer Res..

[122] Dou Z., Ghosh K., Vizioli M.G., Zhu J., Sen P., Wangensteen K.J., Simithy J., Lan Y., Lin Y., Zhou Z. (2017). Cytoplasmic Chromatin Triggers Inflammation in Senescence and Cancer. Nature.

[123] Sun L., Wu J., Du F., Chen X., Chen Z.J. (2013). Cyclic GMP-AMP Synthase Is a Cytosolic DNA Sensor That Activates the Type I Interferon Pathway. Science.

[124] Zhang C., Shang G., Gui X., Zhang X., Bai X., Chen Z.J. (2019). Structural Basis of STING Binding with and Phosphorylation by TBK1. Nature.

[125] Li X.-D., Wu J., Gao D., Wang H., Sun L., Chen Z.J. (2013). Pivotal Roles of CGAS-CGAMP Signaling in Antiviral Defense and Immune Adjuvant Effects. Science.

[126] Ishikawa H., Barber G.N. (2008). STING Is an Endoplasmic Reticulum Adaptor That Facilitates Innate Immune Signalling. Nature.

[127] Yum S., Li M., Fang Y., Chen Z.J. (2021). TBK1 Recruitment to STING Activates Both IRF3 and NF-ΚB That Mediate Immune Defense against Tumors and Viral Infections. Proc. Natl. Acad. Sci. USA.

[128] Brzostek-Racine S., Gordon C., Van Scoy S., Reich N.C. (2011). The DNA Damage Response Induces IFN. J. Immunol..

[129] Parker B.S., Rautela J., Hertzog P.J. (2016). Antitumour Actions of Interferons: Implications for Cancer Therapy. Nat. Rev. Cancer.

[130] Liu H., Zhang H., Wu X., Ma D., Wu J., Wang L., Jiang Y., Fei Y., Zhu C., Tan R. (2018). Nuclear CGAS Suppresses DNA Repair and Promotes Tumorigenesis. Nature.

[131] Bai J., Liu F. (2022). Nuclear CGAS: Sequestration and Beyond. Protein Cell.

[132] Chen H., Chen H., Zhang J., Wang Y., Simoneau A., Yang H., Levine A.S., Zou L., Chen Z., Lan L. (2020). CGAS Suppresses Genomic Instability as a Decelerator of Replication Forks. Sci. Adv..

[133] Zhang Z., Yuan B., Bao M., Lu N., Kim T., Liu Y.-J. (2011). The Helicase DDX41 Senses Intracellular DNA Mediated by the Adaptor STING in Dendritic Cells. Nat. Immunol..

[134] Barber G.N. (2011). STING-Dependent Signaling. Nat. Immunol..

[135] Unterholzner L., Keating S.E., Baran M., Horan K.A., Jensen S.B., Sharma S., Sirois C.M., Jin T., Latz E., Xiao T.S. (2010). IFI16 Is an Innate Immune Sensor for Intracellular DNA. Nat. Immunol..

[136] Cao X. (2016). Self-Regulation and Cross-Regulation of Pattern-Recognition Receptor Signalling in Health and Disease. Nat. Rev. Immunol..

[137] Takaoka A., Wang Z., Choi M.K., Yanai H., Negishi H., Ban T., Lu Y., Miyagishi M., Kodama T., Honda K. (2007). DAI (DLM-1/ZBP1) Is a Cytosolic DNA Sensor and an Activator of Innate Immune Response. Nature.

[138] Hornung V., Ablasser A., Charrel-Dennis M., Bauernfeind F., Horvath G., Caffrey Daniel R., Latz E., Fitzgerald K.A. (2009). AIM2 Recognizes Cytosolic DsDNA and Forms a Caspase-1-Activating Inflammasome with ASC. Nature.

[139] Chiu Y.-H., MacMillan J.B., Chen Z.J. (2009). RNA Polymerase III Detects Cytosolic DNA and Induces Type I Interferons through the RIG-I Pathway. Cell.

[140] Mankan A.K., Schmidt T., Chauhan D., Goldeck M., Höning K., Gaidt M., Kubarenko A.V., Andreeva L., Hopfner K., Hornung V. (2014). Cytosolic RNA:DNA Hybrids Activate the cGAS –STING Axis. EMBO J..

[141] Gasser S., Orsulic S., Brown E.J., Raulet D.H. (2005). The DNA Damage Pathway Regulates Innate Immune System Ligands of the NKG2D Receptor. Nature.

[142] Deng W., Gowen B.G., Zhang L., Wang L., Lau S., Iannello A., Xu J., Rovis T.L., Xiong N., Raulet D.H. (2015). A Shed NKG2D Ligand That Promotes Natural Killer Cell Activation and Tumor Rejection. Science.

[143] Tang K.-F., Ren H., Cao J., Zeng G.-L., Xie J., Chen M., Wang L., He C.-X. (2008). Decreased Dicer Expression Elicits DNA Damage and Up-Regulation of MICA and MICB. J. Cell Biol..

[144] Davalos A.R., Kawahara M., Malhotra G.K., Schaum N., Huang J., Ved U., Beausejour C.M., Coppe J.-P., Rodier F., Campisi J. (2013). P53-Dependent Release of Alarmin HMGB1 Is a Central Mediator of Senescent Phenotypes. J. Cell Biol..

[145] Kang C., Xu Q., Martin T.D., Li M.Z., Demaria M., Aron L., Lu T., Yankner B.A., Campisi J., Elledge S.J. (2015). The DNA Damage Response Induces Inflammation and Senescence by Inhibiting Autophagy of GATA4. Science.

[146] Tchkonia T., Zhu Y., van Deursen J., Campisi J., Kirkland J.L. (2013). Cellular Senescence and the Senescent Secretory Phenotype: Therapeutic Opportunities. J. Clin. Investig..

[147] Coppé J.-P., Desprez P.-Y., Krtolica A., Campisi J. (2010). The Senescence-Associated Secretory Phenotype: The Dark Side of Tumor Suppression. Annu. Rev. Pathol. Mech. Dis..

[148] Campisi J. (2013). Aging, Cellular Senescence, and Cancer. Annu. Rev. Physiol..

[149] Ma J., Setton J., Lee N.Y., Riaz N., Powell S.N. (2018). The Therapeutic Significance of Mutational Signatures from DNA Repair Deficiency in Cancer. Nat. Commun..

[150] Permata T.B.M., Hagiwara Y., Sato H., Yasuhara T., Oike T., Gondhowiardjo S., Held K.D., Nakano T., Shibata A. (2019). Base Excision Repair Regulates PD-L1 Expression in Cancer Cells. Oncogene.

[151] Golan T., O’Kane G.M., Denroche R.E., Raitses-Gurevich M., Grant R.C., Holter S., Wang Y., Zhang A., Jang G.H., Stossel C. (2021). Genomic Features and Classification of Homologous Recombination Deficient Pancreatic Ductal Adenocarcinoma. Gastroenterology.

[152] Jiricny J. (2013). Postreplicative Mismatch Repair. Cold Spring Harb. Perspect. Biol..

[153] Ma X., Dong L., Liu X., Ou K., Yang L. (2022). POLE/POLD1 Mutation and Tumor Immunotherapy. J. Exp. Clin. Cancer Res..

[154] Amodio V., Lamba S., Chilà R., Cattaneo C.M., Mussolin B., Corti G., Rospo G., Berrino E., Tripodo C., Pisati F. (2023). Genetic and Pharmacological Modulation of DNA Mismatch Repair Heterogeneous Tumors Promotes Immune Surveillance. Cancer Cell.

[155] De Mattos-Arruda L., Vazquez M., Finotello F., Lepore R., Porta E., Hundal J., Amengual-Rigo P., Ng C.K.Y., Valencia A., Carrillo J. (2020). Neoantigen Prediction and Computational Perspectives towards Clinical Benefit: Recommendations from the ESMO Precision Medicine Working Group. Ann. Oncol..

[156] Kiyotani K., Chan H.T., Nakamura Y. (2018). Immunopharmacogenomics towards Personalized Cancer Immunotherapy Targeting Neoantigens. Cancer Sci..

[157] Chen F., Zou Z., Du J., Su S., Shao J., Meng F., Yang J., Xu Q., Ding N., Yang Y. (2019). Neoantigen Identification Strategies Enable Personalized Immunotherapy in Refractory Solid Tumors. J. Clin. Investig..

[158] Roudko V., Greenbaum B., Bhardwaj N. (2020). Computational Prediction and Validation of Tumor-Associated Neoantigens. Front. Immunol..

[159] Richters M.M., Xia H., Campbell K.M., Gillanders W.E., Griffith O.L., Griffith M. (2019). Best Practices for Bioinformatic Characterization of Neoantigens for Clinical Utility. Genome Med..

[160] Lu Y.-C., Robbins P.F. (2016). Targeting Neoantigens for Cancer Immunotherapy: Table 1. Int. Immunol..

[161] Dai J., Jiang M., He K., Wang H., Chen P., Guo H., Zhao W., Lu H., He Y., Zhou C. (2021). DNA Damage Response and Repair Gene Alterations Increase Tumor Mutational Burden and Promote Poor Prognosis of Advanced Lung Cancer. Front. Oncol..

[162] Georgoulias G., Zaravinos A. (2022). Genomic Landscape of the Immunogenicity Regulation in Skin Melanomas with Diverse Tumor Mutation Burden. Front. Immunol..

[163] Chen M., Huang B., Zhu L., Wang Q., Pang Y., Cheng M., Lian H., Liu M., Zhao K., Xu S. (2022). DNA Damage Response Evaluation Provides Novel Insights for Personalized Immunotherapy in Glioma. Front. Immunol..

[164] Lou S., Wang Y., Zhang J., Yin X., Zhang Y., Wang Y., Xue Y. (2022). Patient-Level DNA Damage Repair Pathway Profiles and Anti-Tumor Immunity for Gastric Cancer. Front. Immunol..

[165] Zhang T., Zheng S., Liu Y., Li X., Wu J., Sun Y., Liu G. (2021). DNA Damage Response and PD-1/PD-L1 Pathway in Ovarian Cancer. DNA Repair.

[166] Hutchinson L. (2017). Aneuploidy and Immune Evasion—A Biomarker of Response. Nat. Rev. Clin. Oncol..

[167] Spurr L.F., Weichselbaum R.R., Pitroda S.P. (2022). Tumor Aneuploidy Predicts Survival Following Immunotherapy across Multiple Cancers. Nat. Genet..

[168] Spurr L.F., Martinez C.A., Kang W., Chen M., Zha Y., Hseu R., Gutiontov S.I., Turchan W.T., Lynch C.M., Pointer K.B. (2022). Highly Aneuploid Non-Small Cell Lung Cancer Shows Enhanced Responsiveness to Concurrent Radiation and Immune Checkpoint Blockade. Nat. Cancer.

[169] Zanetti M. (2017). Chromosomal Chaos Silences Immune Surveillance. Science.

[170] Davoli T., Uno H., Wooten E.C., Elledge S.J. (2017). Tumor Aneuploidy Correlates with Markers of Immune Evasion and with Reduced Response to Immunotherapy. Science.

[171] Sloan E.A., Ring K.L., Willis B.C., Modesitt S.C., Mills A.M. (2017). PD-L1 Expression in Mismatch Repair-Deficient Endometrial Carcinomas, Including Lynch Syndrome-Associated and MLH1 Promoter Hypermethylated Tumors. Am. J. Surg. Pathol..

[172] Favier A., Varinot J., Uzan C., Duval A., Brocheriou I., Canlorbe G. (2022). The Role of Immunohistochemistry Markers in Endometrial Cancer with Mismatch Repair Deficiency: A Systematic Review. Cancers.

[173] Ramchander N.C., Ryan N.A.J., Walker T.D.J., Harries L., Bolton J., Bosse T., Evans D.G., Crosbie E.J. (2020). Distinct Immunological Landscapes Characterize Inherited and Sporadic Mismatch Repair Deficient Endometrial Cancer. Front. Immunol..

[174] Mills A.M., Dill E.A., Moskaluk C.A., Dziegielewski J., Bullock T.N., Dillon P.M. (2018). The Relationship Between Mismatch Repair Deficiency and PD-L1 Expression in Breast Carcinoma. Am. J. Surg. Pathol..

[175] Dong H., Strome S.E., Salomao D.R., Tamura H., Hirano F., Flies D.B., Roche P.C., Lu J., Zhu G., Tamada K. (2002). Tumor-Associated B7-H1 Promotes T-Cell Apoptosis: A Potential Mechanism of Immune Evasion. Nat. Med..

[176] Garcia-Diaz A., Shin D.S., Moreno B.H., Saco J., Escuin-Ordinas H., Rodriguez G.A., Zaretsky J.M., Sun L., Hugo W., Wang X. (2017). Interferon Receptor Signaling Pathways Regulating PD-L1 and PD-L2 Expression. Cell Rep..

[177] Chabanon R.M., Muirhead G., Krastev D.B., Adam J., Morel D., Garrido M., Lamb A., Hénon C., Dorvault N., Rouanne M. (2019). PARP Inhibition Enhances Tumor Cell–Intrinsic Immunity in ERCC1-Deficient Non–Small Cell Lung Cancer. J. Clin. Investig..

[178] Wang Y., Zheng K., Xiong H., Huang Y., Chen X., Zhou Y., Qin W., Su J., Chen R., Qiu H. (2021). PARP Inhibitor Upregulates PD-L1 Expression and Provides a New Combination Therapy in Pancreatic Cancer. Front. Immunol..

[179] Sato H., Jeggo P.A., Shibata A. (2019). Regulation of Programmed Death-ligand 1 Expression in Response to DNA Damage in Cancer Cells: Implications for Precision Medicine. Cancer Sci..

[180] Sato H., Niimi A., Yasuhara T., Permata T.B.M., Hagiwara Y., Isono M., Nuryadi E., Sekine R., Oike T., Kakoti S. (2017). DNA Double-Strand Break Repair Pathway Regulates PD-L1 Expression in Cancer Cells. Nat. Commun..

[181] Mouw K.W., Konstantinopoulos P.A. (2018). From Checkpoint to Checkpoint: DNA Damage ATR/Chk1 Checkpoint Signalling Elicits PD-L1 Immune Checkpoint Activation. Br. J. Cancer.

[182] Parkes E.E., Walker S.M., Taggart L.E., McCabe N., Knight L.A., Wilkinson R., McCloskey K.D., Buckley N.E., Savage K.I., Salto-Tellez M. (2017). Activation of STING-Dependent Innate Immune Signaling By S-Phase-Specific DNA Damage in Breast Cancer. J. Natl. Cancer Inst..

[183] Shi C., Qin K., Lin A., Jiang A., Cheng Q., Liu Z., Zhang J., Luo P. (2022). The Role of DNA Damage Repair (DDR) System in Response to Immune Checkpoint Inhibitor (ICI) Therapy. J. Exp. Clin. Cancer Res..

[184] Liu J., Hamrouni A., Wolowiec D., Coiteux V., Kuliczkowski K., Hetuin D., Saudemont A., Quesnel B. (2007). Plasma Cells from Multiple Myeloma Patients Express B7-H1 (PD-L1) and Increase Expression after Stimulation with IFN-γ and TLR Ligands via a MyD88-, TRAF6-, and MEK-Dependent Pathway. Blood.

[185] Apetoh L., Ghiringhelli F., Tesniere A., Obeid M., Ortiz C., Criollo A., Mignot G., Maiuri M.C., Ullrich E., Saulnier P. (2007). Toll-like Receptor 4–Dependent Contribution of the Immune System to Anticancer Chemotherapy and Radiotherapy. Nat. Med..

[186] Blank C., Gajewski T.F., Mackensen A. (2005). Interaction of PD-L1 on Tumor Cells with PD-1 on Tumor-Specific T Cells as a Mechanism of Immune Evasion: Implications for Tumor Immunotherapy. Cancer Immunol. Immunother..

[187] Iwai Y., Ishida M., Tanaka Y., Okazaki T., Honjo T., Minato N. (2002). Involvement of PD-L1 on Tumor Cells in the Escape from Host Immune System and Tumor Immunotherapy by PD-L1 Blockade. Proc. Natl. Acad. Sci. USA.

[188] Kajiwara Y., Tazawa H., Yamada M., Kanaya N., Fushimi T., Kikuchi S., Kuroda S., Ohara T., Noma K., Yoshida R. (2022). Oncolytic Virus-Mediated Reducing of Myeloid-Derived Suppressor Cells Enhances the Efficacy of PD-L1 Blockade in Gemcitabine-Resistant Pancreatic Cancer. Cancer Immunol. Immunother..

[189] Al Nabhani S., Al Harthy A., Al Riyami M., Al Sinawi S., Al Rashdi A., Al Husseni S., Kumar S. (2022). Programmed Death-Ligand 1 (PD-L1) Expression in Bladder Cancer and Its Correlation with Tumor Grade, Stage, and Outcome. Oman Med. J..

[190] Tu X., Qin B., Zhang Y., Zhang C., Kahila M., Nowsheen S., Yin P., Yuan J., Pei H., Li H. (2019). PD-L1 (B7-H1) Competes with the RNA Exosome to Regulate the DNA Damage Response and Can Be Targeted to Sensitize to Radiation or Chemotherapy. Mol. Cell.

[191] Freeman G.J., Long A.J., Iwai Y., Bourque K., Chernova T., Nishimura H., Fitz L.J., Malenkovich N., Okazaki T., Byrne M.C. (2000). Engagement of the Pd-1 Immunoinhibitory Receptor by a Novel B7 Family Member Leads to Negative Regulation of Lymphocyte Activation. J. Exp. Med..

[192] Brunner S., Herndler-Brandstetter D., Arnold C.R., Wiegers G.J., Villunger A., Hackl M., Grillari J., Moreno-Villanueva M., Bürkle A., Grubeck-Loebenstein B. (2012). Upregulation of MiR-24 Is Associated with a Decreased DNA Damage Response upon Etoposide Treatment in Highly Differentiated CD8 ^+^ T Cells Sensitizing Them to Apoptotic Cell Death. Aging Cell.

[193] Yan Q., Zhang B., Ling X., Zhu B., Mei S., Yang H., Zhang D., Huo J., Zhao Z. (2022). CTLA-4 Facilitates DNA Damage–Induced Apoptosis by Interacting with PP2A. Front. Cell Dev. Biol..

[194] Ryan A.E., Shanahan F., O’Connell J., Houston A.M. (2005). Addressing the “Fas Counterattack” Controversy: Blocking Fas Ligand Expression Suppresses Tumor Immune Evasion of Colon Cancer In Vivo. Cancer Res..

[195] Zhu J., Powis de Tenbossche C.G., Cané S., Colau D., van Baren N., Lurquin C., Schmitt-Verhulst A.-M., Liljeström P., Uyttenhove C., Van den Eynde B.J. (2017). Resistance to Cancer Immunotherapy Mediated by Apoptosis of Tumor-Infiltrating Lymphocytes. Nat. Commun..

[196] Upadhyay R., Boiarsky J.A., Pantsulaia G., Svensson-Arvelund J., Lin M.J., Wroblewska A., Bhalla S., Scholler N., Bot A., Rossi J.M. (2021). A Critical Role for Fas-Mediated Off-Target Tumor Killing in T-Cell Immunotherapy. Cancer Discov..

[197] Raats D.A., Frenkel N., van Schelven S.J., Rinkes I.H., Laoukili J., Kranenburg O. (2017). CD95 Ligand Induces Senescence in Mismatch Repair-Deficient Human Colon Cancer via Chronic Caspase-Mediated Induction of DNA Damage. Cell Death Dis..

[198] Gullickson P., Xu Y.W., Niedernhofer L.J., Thompson E.L., Yousefzadeh M.J. (2022). The Role of DNA Repair in Immunological Diversity: From Molecular Mechanisms to Clinical Ramifications. Front. Immunol..

[199] Bednarski J.J., Sleckman B.P. (2019). At the Intersection of DNA Damage and Immune Responses. Nat. Rev. Immunol..

[200] Hoolehan W., Harris J.C., Byrum J.N., Simpson D.A., Rodgers K.K. (2022). An Updated Definition of V(D)J Recombination Signal Sequences Revealed by High-Throughput Recombination Assays. Nucleic Acids Res..

[201] Alt F.W., Zhang Y., Meng F.-L., Guo C., Schwer B. (2013). Mechanisms of Programmed DNA Lesions and Genomic Instability in the Immune System. Cell.

[202] Johnston R., Mathias B., Crowley S.J., Schmidt H.A., White L.S., Mosammaparast N., Green A.M., Bednarski J.J. (2023). Nuclease-independent Functions of RAG1 Direct Distinct DNA Damage Responses in B Cells. EMBO Rep..

[203] Picard C., Bobby Gaspar H., Al-Herz W., Bousfiha A., Casanova J.-L., Chatila T., Crow Y.J., Cunningham-Rundles C., Etzioni A., Franco J.L. (2018). International Union of Immunological Societies: 2017 Primary Immunodeficiency Diseases Committee Report on Inborn Errors of Immunity. J. Clin. Immunol..

[204] Niewolik D., Schwarz K. (2022). Physical ARTEMIS:DNA-PKcs Interaction Is Necessary for V(D)J Recombination. Nucleic Acids Res..

[205] Thientosapol E.S., Sharbeen G., Lau K.K.E., Bosnjak D., Durack T., Stevanovski I., Weninger W., Jolly C.J. (2016). Proximity to AGCT Sequences Dictates MMR-Independent versus MMR-Dependent Mechanisms for AID-Induced Mutation via UNG2. Nucleic Acids Res..

[206] Safavi S., Larouche A., Zahn A., Patenaude A.-M., Domanska D., Dionne K., Rognes T., Dingler F., Kang S.-K., Liu Y. (2021). The Uracil-DNA Glycosylase UNG Protects the Fitness of Normal and Cancer B Cells Expressing AID. NAR Cancer.

[207] Roco J.A., Mesin L., Binder S.C., Nefzger C., Gonzalez-Figueroa P., Canete P.F., Ellyard J., Shen Q., Robert P.A., Cappello J. (2019). Class-Switch Recombination Occurs Infrequently in Germinal Centers. Immunity.

[208] Weitering T.J., Takada S., Weemaes C.M.R., van Schouwenburg P.A., van der Burg M. (2021). ATM: Translating the DNA Damage Response to Adaptive Immunity. Trends Immunol..

[209] Bahjat M., Guikema J. (2017). The Complex Interplay between DNA Injury and Repair in Enzymatically Induced Mutagenesis and DNA Damage in B Lymphocytes. Int. J. Mol. Sci..

[210] Stratigopoulou M., van Dam T.P., Guikema J.E.J. (2020). Base Excision Repair in the Immune System: Small DNA Lesions with Big Consequences. Front. Immunol..

[211] Rotte A., Jin J.Y., Lemaire V. (2018). Mechanistic Overview of Immune Checkpoints to Support the Rational Design of Their Combinations in Cancer Immunotherapy. Ann. Oncol..

[212] André T., Tougeron D., Piessen G., de la Fouchardière C., Louvet C., Adenis A., Jary M., Tournigand C., Aparicio T., Desrame J. (2023). Neoadjuvant Nivolumab Plus Ipilimumab and Adjuvant Nivolumab in Localized Deficient Mismatch Repair/Microsatellite Instability–High Gastric or Esophagogastric Junction Adenocarcinoma: The GERCOR NEONIPIGA Phase II Study. J. Clin. Oncol..

[213] André T., Shiu K.-K., Kim T.W., Jensen B.V., Jensen L.H., Punt C., Smith D., Garcia-Carbonero R., Benavides M., Gibbs P. (2020). Pembrolizumab in Microsatellite-Instability–High Advanced Colorectal Cancer. N. Engl. J. Med..

[214] Overman M.J., McDermott R., Leach J.L., Lonardi S., Lenz H.-J., Morse M.A., Desai J., Hill A., Axelson M., Moss R.A. (2017). Nivolumab in Patients with Metastatic DNA Mismatch Repair-Deficient or Microsatellite Instability-High Colorectal Cancer (CheckMate 142): An Open-Label, Multicentre, Phase 2 Study. Lancet Oncol..

[215] Sahin I.H., Akce M., Alese O., Shaib W., Lesinski G.B., El-Rayes B., Wu C. (2019). Immune Checkpoint Inhibitors for the Treatment of MSI-H/MMR-D Colorectal Cancer and a Perspective on Resistance Mechanisms. Br. J. Cancer.

[216] Jiang M., Jia K., Wang L., Li W., Chen B., Liu Y., Wang H., Zhao S., He Y., Zhou C. (2021). Alterations of DNA Damage Response Pathway: Biomarker and Therapeutic Strategy for Cancer Immunotherapy. Acta Pharm. Sin. B.

[217] Wang D.-R., Wu X.-L., Sun Y.-L. (2022). Therapeutic Targets and Biomarkers of Tumor Immunotherapy: Response versus Non-Response. Signal Transduct. Target. Ther..

[218] Grimaldi A., Cammarata I., Martire C., Focaccetti C., Piconese S., Buccilli M., Mancone C., Buzzacchino F., Berrios J.R.G., D’Alessandris N. (2020). Combination of Chemotherapy and PD-1 Blockade Induces T Cell Responses to Tumor Non-Mutated Neoantigens. Commun. Biol..

[219] Donlon N.E., Power R., Hayes C., Reynolds J.V., Lysaght J. (2021). Radiotherapy, Immunotherapy, and the Tumour Microenvironment: Turning an Immunosuppressive Milieu into a Therapeutic Opportunity. Cancer Lett..

[220] Ye Z., Shi Y., Lees-Miller S.P., Tainer J.A. (2021). Function and Molecular Mechanism of the DNA Damage Response in Immunity and Cancer Immunotherapy. Front. Immunol..

[221] Brown J.S., O’Carrigan B., Jackson S.P., Yap T.A. (2017). Targeting DNA Repair in Cancer: Beyond PARP Inhibitors. Cancer Discov..

[222] Cetin B., Wabl C.A., Gumusay O. (2020). The DNA Damaging Revolution. Crit. Rev. Oncol. Hematol..

[223] Farmer H., McCabe N., Lord C.J., Tutt A.N.J., Johnson D.A., Richardson T.B., Santarosa M., Dillon K.J., Hickson I., Knights C. (2005). Targeting the DNA Repair Defect in BRCA Mutant Cells as a Therapeutic Strategy. Nature.

[224] Ledermann J.A., Harter P., Gourley C., Friedlander M., Vergote I., Rustin G., Scott C., Meier W., Shapira-Frommer R., Safra T. (2016). Overall Survival in Patients with Platinum-Sensitive Recurrent Serous Ovarian Cancer Receiving Olaparib Maintenance Monotherapy: An Updated Analysis from a Randomised, Placebo-Controlled, Double-Blind, Phase 2 Trial. Lancet Oncol..

[225] Ledermann J., Harter P., Gourley C., Friedlander M., Vergote I., Rustin G., Scott C.L., Meier W., Shapira-Frommer R., Safra T. (2014). Olaparib Maintenance Therapy in Patients with Platinum-Sensitive Relapsed Serous Ovarian Cancer: A Preplanned Retrospective Analysis of Outcomes by BRCA Status in a Randomised Phase 2 Trial. Lancet Oncol..

[226] Ledermann J., Harter P., Gourley C., Friedlander M., Vergote I., Rustin G., Scott C., Meier W., Shapira-Frommer R., Safra T. (2012). Olaparib Maintenance Therapy in Platinum-Sensitive Relapsed Ovarian Cancer. N. Engl. J. Med..

[227] Higuchi T., Flies D.B., Marjon N.A., Mantia-Smaldone G., Ronner L., Gimotty P.A., Adams S.F. (2015). CTLA-4 Blockade Synergizes Therapeutically with PARP Inhibition in BRCA1-Deficient Ovarian Cancer. Cancer Immunol. Res..

[228] Ding L., Kim H.-J., Wang Q., Kearns M., Jiang T., Ohlson C.E., Li B.B., Xie S., Liu J.F., Stover E.H. (2018). PARP Inhibition Elicits STING-Dependent Antitumor Immunity in Brca1-Deficient Ovarian Cancer. Cell Rep..

[229] Lee E.K., Konstantinopoulos P.A. (2019). Combined PARP and Immune Checkpoint Inhibition in Ovarian Cancer. Trends Cancer.

[230] Shen J., Zhao W., Ju Z., Wang L., Peng Y., Labrie M., Yap T.A., Mills G.B., Peng G. (2019). PARPi Triggers the STING-Dependent Immune Response and Enhances the Therapeutic Efficacy of Immune Checkpoint Blockade Independent of BRCAness. Cancer Res..

[231] Ramalingam S.S., Thara E., Awad M.M., Dowlati A., Haque B., Stinchcombe T.E., Dy G.K., Spigel D.R., Lu S., Iyer Singh N. (2022). JASPER: Phase 2 Trial of First-line Niraparib plus Pembrolizumab in Patients with Advanced Non–Small Cell Lung Cancer. Cancer.

[232] Vinayak S., Tolaney S.M., Schwartzberg L., Mita M., McCann G., Tan A.R., Wahner-Hendrickson A.E., Forero A., Anders C., Wulf G.M. (2019). Open-Label Clinical Trial of Niraparib Combined with Pembrolizumab for Treatment of Advanced or Metastatic Triple-Negative Breast Cancer. JAMA Oncol..

[233] Houl J.H., Ye Z., Brosey C.A., Balapiti-Modarage L.P.F., Namjoshi S., Bacolla A., Laverty D., Walker B.L., Pourfarjam Y., Warden L.S. (2019). Selective Small Molecule PARG Inhibitor Causes Replication Fork Stalling and Cancer Cell Death. Nat. Commun..

[234] Fathers C., Drayton R.M., Solovieva S., Bryant H.E. (2012). Inhibition of Poly(ADP-Ribose) Glycohydrolase (PARG) Specifically Kills BRCA2-Deficient Tumor Cells. Cell Cycle.

[235] Yap T.A., Krebs M.G., Postel-Vinay S., Bang Y.J., El-Khoueiry A., Abida W., Harrington K., Sundar R., Carter L., Castanon-Alvarez E. (2016). Phase I Modular Study of AZD6738, a Novel Oral, Potent and Selective Ataxia Telangiectasia Rad3-Related (ATR) Inhibitor in Combination (Combo) with Carboplatin, Olaparib or Durvalumab in Patients (Pts) with Advanced Cancers. Eur. J. Cancer.

[236] Morales-Juarez D.A., Jackson S.P. (2022). Clinical Prospects of WRN Inhibition as a Treatment for MSI Tumours. Npj Precis. Oncol..

[237] Kelderman S., Schumacher T.N., Kvistborg P. (2015). Mismatch Repair-Deficient Cancers Are Targets for Anti-PD-1 Therapy. Cancer Cell.

[238] Diaz L.A., Le D.T. (2015). PD-1 Blockade in Tumors with Mismatch-Repair Deficiency. N. Engl. J. Med..

[239] Diaz L.A., Shiu K.-K., Kim T.-W., Jensen B.V., Jensen L.H., Punt C., Smith D., Garcia-Carbonero R., Benavides M., Gibbs P. (2022). Pembrolizumab versus Chemotherapy for Microsatellite Instability-High or Mismatch Repair-Deficient Metastatic Colorectal Cancer (KEYNOTE-177): Final Analysis of a Randomised, Open-Label, Phase 3 Study. Lancet Oncol..

[240] Romero D. (2021). New First-Line Therapy for DMMR/MSI-H CRC. Nat. Rev. Clin. Oncol..

[241] Zhao P., Li L., Jiang X., Li Q. (2019). Mismatch Repair Deficiency/Microsatellite Instability-High as a Predictor for Anti-PD-1/PD-L1 Immunotherapy Efficacy. J. Hematol. Oncol..

[242] Durbin R.P. (1975). Letter: Acid Secretion by Gastric Mucous Membrane. Am. J. Physiol..

[243] Le D.T., Uram J.N., Wang H., Bartlett B.R., Kemberling H., Eyring A.D., Skora A.D., Luber B.S., Azad N.S., Laheru D. (2015). PD-1 Blockade in Tumors with Mismatch-Repair Deficiency. N. Engl. J. Med..

[244] Hellmann M.D., Ciuleanu T.-E., Pluzanski A., Lee J.S., Otterson G.A., Audigier-Valette C., Minenza E., Linardou H., Burgers S., Salman P. (2018). Nivolumab plus Ipilimumab in Lung Cancer with a High Tumor Mutational Burden. N. Engl. J. Med..

[245] (2018). High TMB Predicts Immunotherapy Benefit. Cancer Discov..

[246] Killock D. (2018). Nivolumab–Ipilimumab—Exploiting the Mutation Burden of NSCLCs. Nat. Rev. Clin. Oncol..

[247] Ready N., Hellmann M.D., Awad M.M., Otterson G.A., Gutierrez M., Gainor J.F., Borghaei H., Jolivet J., Horn L., Mates M. (2019). First-Line Nivolumab Plus Ipilimumab in Advanced Non–Small-Cell Lung Cancer (CheckMate 568): Outcomes by Programmed Death Ligand 1 and Tumor Mutational Burden as Biomarkers. J. Clin. Oncol..

[248] Chalmers Z.R., Connelly C.F., Fabrizio D., Gay L., Ali S.M., Ennis R., Schrock A., Campbell B., Shlien A., Chmielecki J. (2017). Analysis of 100,000 Human Cancer Genomes Reveals the Landscape of Tumor Mutational Burden. Genome Med..

[249] Ding K., He Y., Wei J., Fu S., Wang J., Chen Z., Zhang H., Qu Y., Liang K., Gong X. (2022). A Score of DNA Damage Repair Pathway with the Predictive Ability for Chemotherapy and Immunotherapy Is Strongly Associated with Immune Signaling Pathway in Pan-Cancer. Front. Immunol..

[250] Chen Y., Wang X., Deng X., Zhang Y., Liao R., Li Y., Yang H., Chen K. (2021). DNA Damage Repair Status Predicts Opposite Clinical Prognosis Immunotherapy and Non-Immunotherapy in Hepatocellular Carcinoma. Front. Immunol..

[251] Bruand M., Barras D., Mina M., Ghisoni E., Morotti M., Lanitis E., Fahr N., Desbuisson M., Grimm A., Zhang H. (2021). Cell-Autonomous Inflammation of BRCA1-Deficient Ovarian Cancers Drives Both Tumor-Intrinsic Immunoreactivity and Immune Resistance via STING. Cell Rep..

